# Author Correction: CITED2 is a druggable epigenetic switch coupling neuronal maturation to regenerative decline

**DOI:** 10.1038/s44321-026-00442-4

**Published:** 2026-05-08

**Authors:** Franziska Müller, Eilidh McLachlan, Ana Catarina Costa, Jia Qu, Bishal Shrestha, Zheng Wang, Francesco De Virgiliis, Thomas Haynes Hutson, Luming Zhou, Guiping Kong, Jessica S Chadwick, Paolo La Montanara, Zhulin Yuan, Nejc Haberman, Monica M Sousa, Ilaria Palmisano, Simone Di Giovanni

**Affiliations:** 1 Division of Neuroscience, Department of Brain Sciences, Imperial College London, London, W12 0NN UK; 2 Nerve Regeneration Group, Instituto de Biologia Molecular e Celular (IBMC), Instituto de Investigação e Inovação em Saúde (i3S), University of Porto, Porto, 4200-135 Portugal; 3 Department of Neuroscience, Ohio State University College of Medicine, Columbus, OH 43210 USA; 4 Department of Computer Science, University of Miami, Coral Gables, FL 33124-4245 USA; 5 Department of Pathology and Immunology, University of Geneva, Geneva, 1211 Switzerland; 6 The Wyss Center for Bio and Neuroengineering in Geneva, Chemin des Mines 9, Geneva, 1202 Switzerland

## Abstract

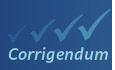

**Correction to:** *EMBO Molecular Medicine* (2026) 18:1174–1201. 10.1038/s44321-026-00385-w | Published online 23 February 2026

**14th author name is corrected**.

The name of the 14th author has been corrected from Nejc Habermann to **Nejc Haberman**.

