# Supplementary material for: CITED2 is a druggable epigenetic switch coupling neuronal maturation to regenerative decline
Source: EMBO Mol Med. 2026 Feb 23;18(4):1174–201. doi: 10.1038/s44321-026-00385-w (PMC13083982; doi:10.1038/s44321-026-00385-w)
Supplement: Supplementary file 1 — Appendix [file 44321_2026_385_MOESM1_ESM.pdf]

APPENDIX

Table of Contents

SUPPLEMENTARY FIGURES

Appendix Figure S1………………………………………………………………………………page 2

Appendix Figure S2………………………………………………………………………………page 3

Appendix Figure S3………………………………………………………………………………page 4

Appendix Figure S4………………………………………………………………………………page 5

Appendix Figure S5………………………………………………………………………………page 6

Appendix Figure S6………………………………………………………………………………page 7

Appendix Figure S7………………………………………………………………………………page 9

SUPPLEMENTARY TABLES

Appendix Table S1…………………………………………………………………………………page 10

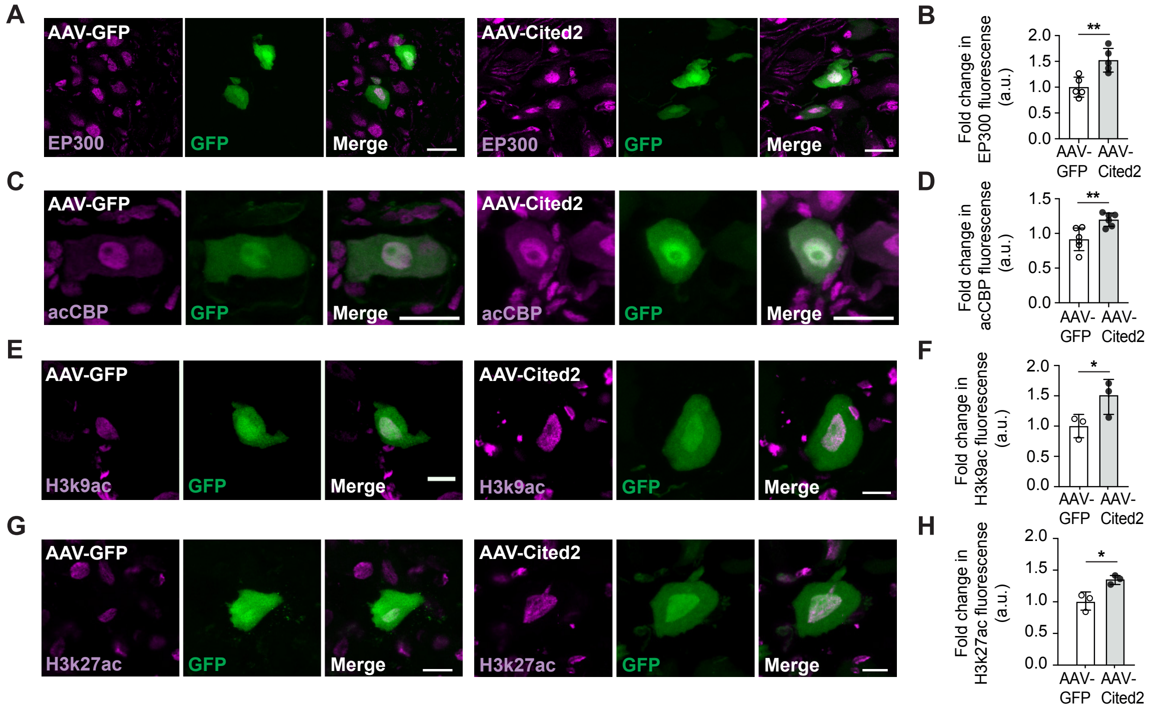

Appendix Figure S1. CITED2 overexpression increases EP300, acCBP and histone H3 acetylation six weeks post-SCI. (A-B) EP300 immunostaining (magenta) in DRG neurons after AAV-GFP or AAV- Cited2-GFP overexpression six weeks post-SCI (p<0.01 by t-test, *n*=5 independent biological replicates). **(C-D)** Acetylated CBP (acCBP) immunostaining (magenta) in DRG neurons after AAV-GFP or AAV-Cited2-GFP overexpression six weeks post-SCI (p<0.01 by t-test, *n*=5 independent biological replicates). **(E-F)** H3k9ac immunostaining (magenta) in DRG neurons after AAV-GFP or AAV-Cited2-GFP overexpression six weeks post-SCI (p<0.05 by t-test, *n*=3 independent biological replicates). **(G-H)** H3k27ac immunostaining (magenta) in DRG neurons after AAV-GFP or AAV-Cited2-GFP overexpression six weeks post-SCI (p<0.05 by t-test, *n*=3 independent biological replicates). DRG neurons were analyzed throughout the thickness of the sciatic DRG. Scale bar: 20µm. *Related to Figure 4.*

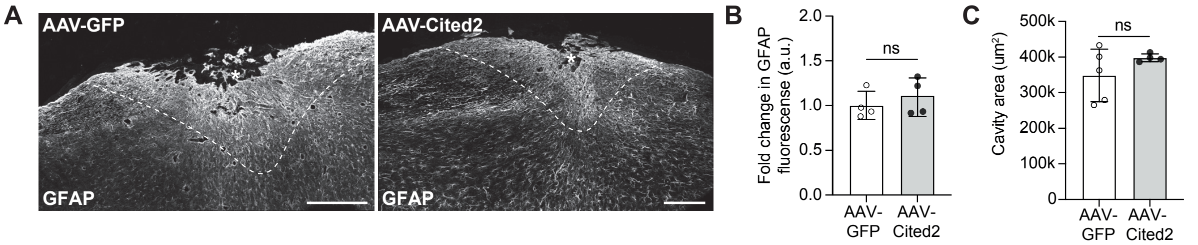

Appendix Figure S2. Cited2 overexpression neither affects GFAP expression nor lesion size. (A-B) GFAP intensity (grey) around SCI site (white asterisk) and cavity size (dotted line) after AAV-GFP or AAV-Cited2-GFP overexpression six weeks post-SCI with quantification of fold change in GFAP intensity (t-test, *n*=4 independent biological replicates). **(C)** Quantification of cavity size in AAV-GFP and AAV-Cited2-GFP overexpressed mice (t-test, *n*=4 and 5 b independent biological replicates). Scale bar: 200 µm. *Related to Figure 4.*

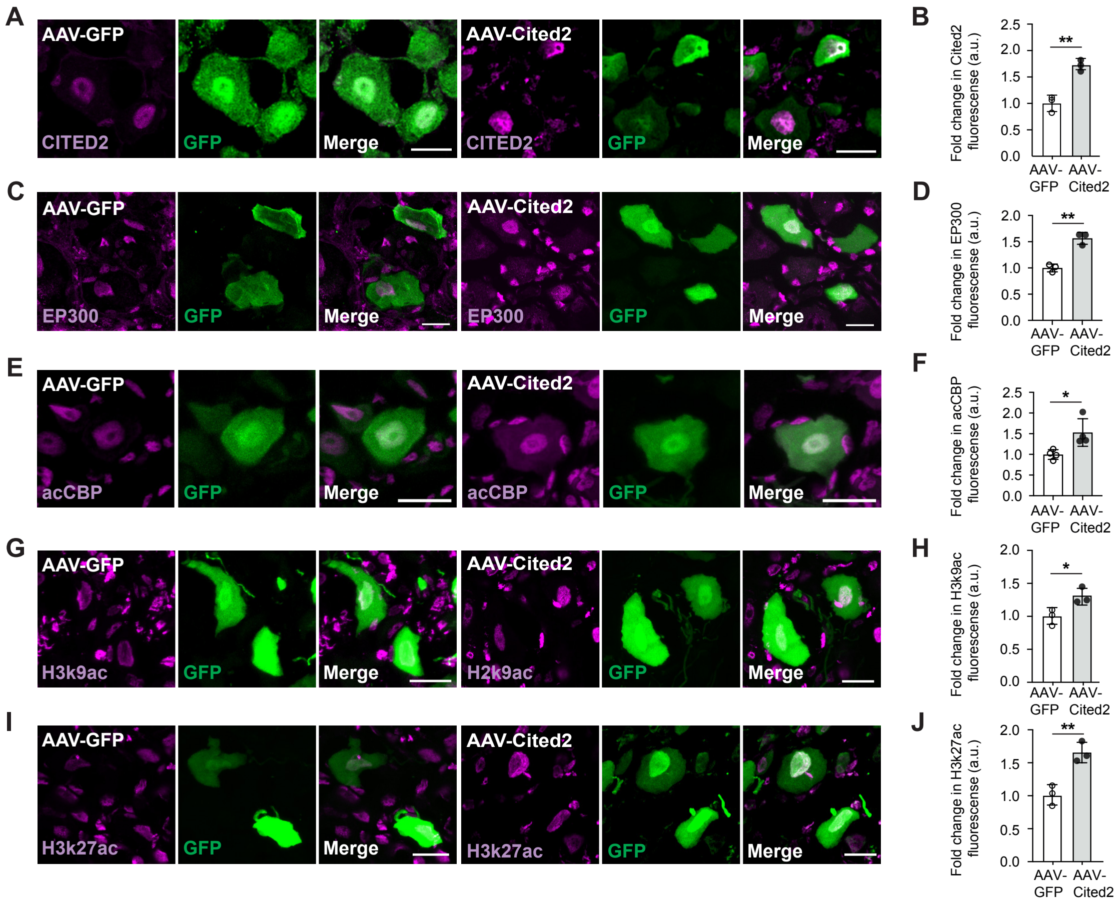

Appendix Figure S3. Cited2 overexpression promotes Cited2, EP300, acCBP expression and histone H3 acetylation post-SCI. (A-B) CITED2 immunostaining (magenta) in DRG neurons after AAV-GFP or AAV-Cited2-GFP overexpression 24 hours post-SCI ($p<0.001$ by t-test, *n*=3 independent biological replicates). **(C-D)** EP300 immunostaining (magenta) in DRG neurons after AAV-GFP or AAV-Cited2-GFP overexpression 24 hours post-SCI ($p<0.001$ by t-test, n=3 independent biological replicates). **(E-F)** AcCBP immunostaining (magenta) in DRG neurons after AAV-GFP or AAV-Cited2-GFP overexpression 24 hours post-SCI ($p<0.05$ by t-test, n=4 independent biological replicates). **(G-H)** H3k9ac immunostaining (magenta) in DRG neurons after AAV-GFP or AAV-Cited2-GFP overexpression 24 hours post-SCI. ($p<0.05$ by t-test, *n*=3 independent biological replicates). **(I-J)** H3k27ac immunostaining (magenta) in DRG neurons after AAV-GFP or AAV-Cited2-GFP overexpression 24 hours post-SCI ($p<0.001$ by t-test, *n*=3 independent biological replicates). DRG neurons were analyzed throughout the thickness of the sciatic DRG. Scale bar: 20 µm. *Related to Figure 5.*

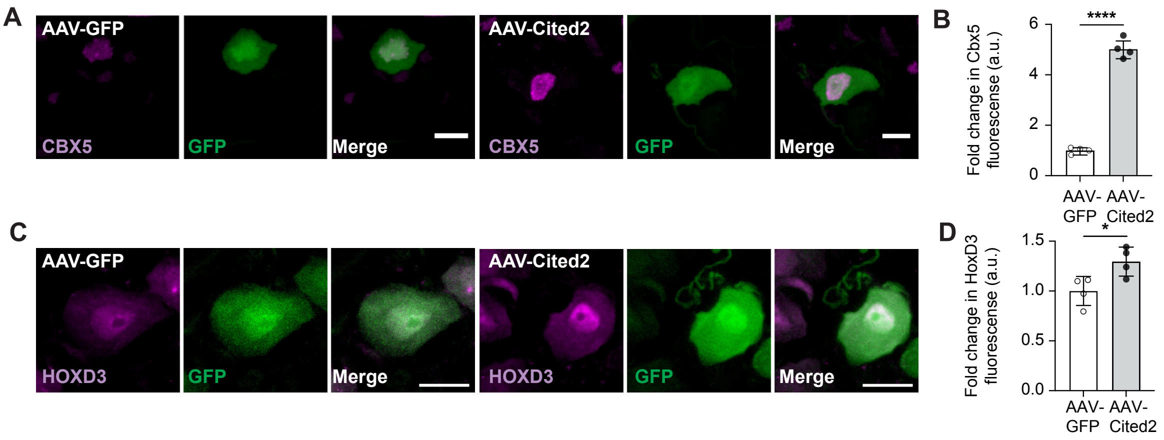

Appendix Figure S4. Cited2 overexpression increases CBX5 and HoxD3 expression post-SCI. (A-B) CBX5 immunostaining (magenta) in DRG neurons after AAV-GFP or AAV-Cited2-GFP overexpression 24 hours post-SCI ($p<0.0001$ by t-test, *n*=4 independent biological replicates). **(C-D)** HOXD3 immunostaining (magenta) in DRG neurons after AAV-GFP or AAV-Cited2-GFP overexpression 24 hours post-SCI ($p<0.05$ by *t-test*, *n*=4 independent biological replicates). DRG neurons were analyzed throughout the thickness of the sciatic DRG. *Related to Figure 5.*

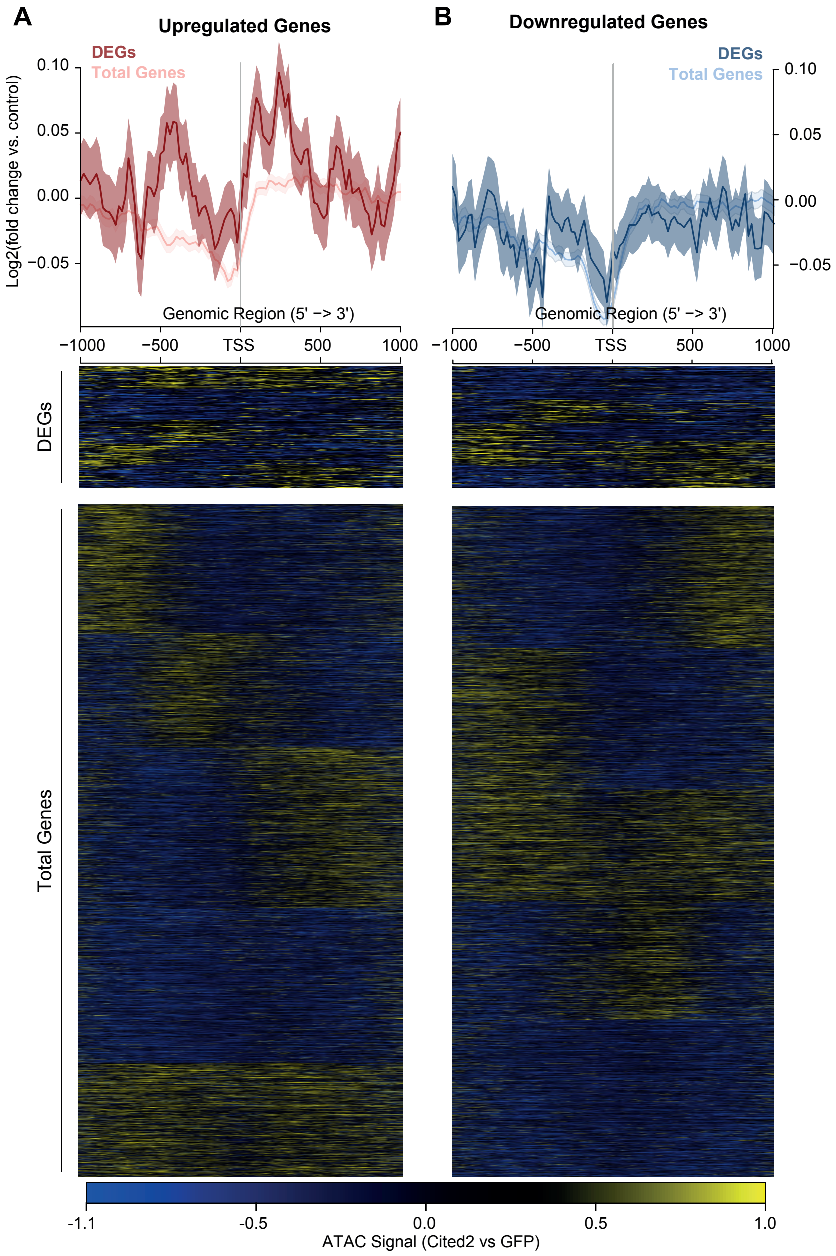

Appendix Figure S5. ATAC signal and gene expression signatures following Cited2 overexpression after SCI. (A-B) ATAC-seq signal density plots and heatmaps for differentially (DEGs; p<0.05) up (A) and downregulated (B) or total genes after CITED2 overexpression versus GFP control at genomic regions 1kb upstream and downstream of the transcription start site (TSS).

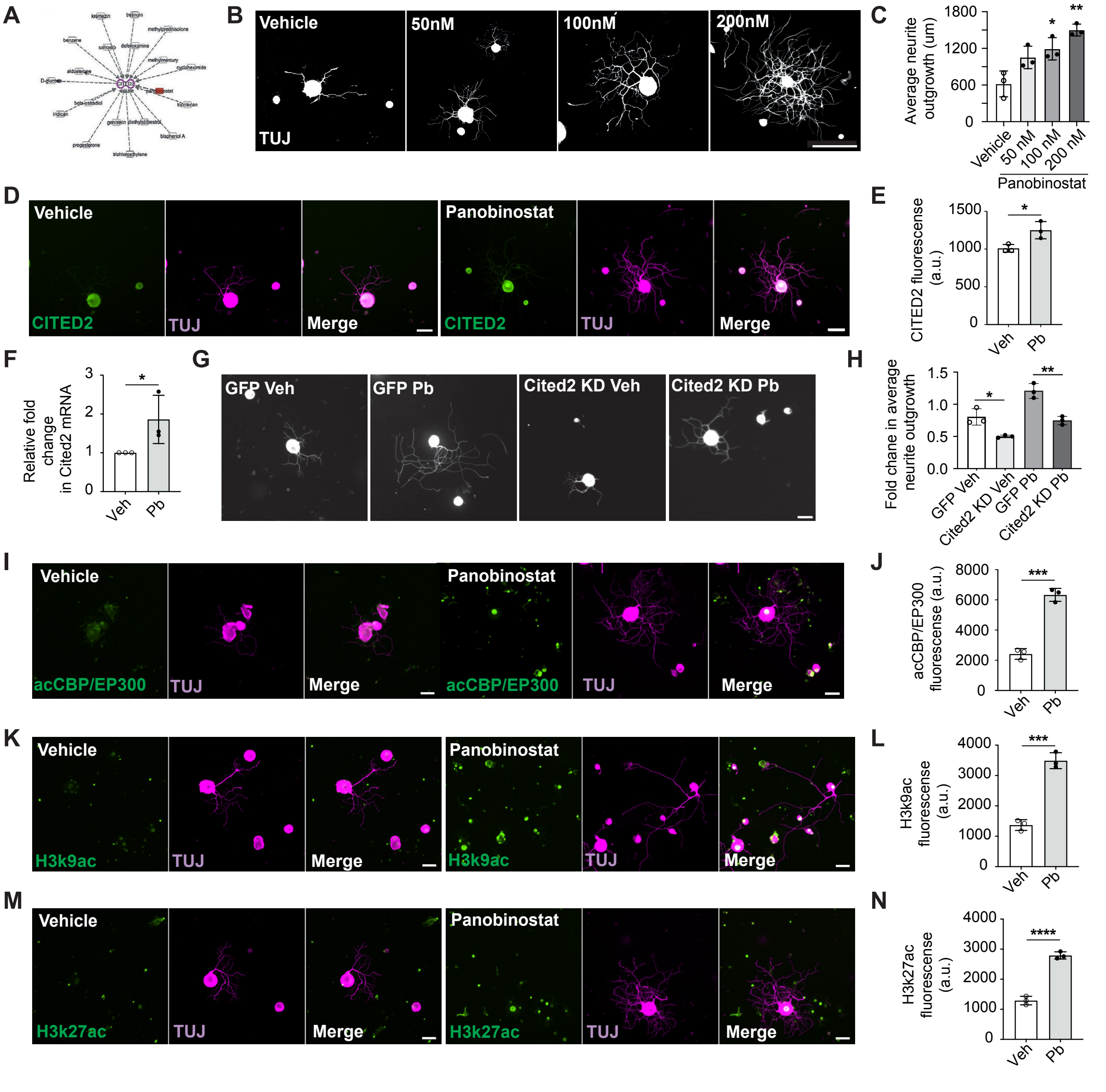

Appendix Figure S6. Panobinostat delivery to cultured DRG neurons promotes Cited2-dependent neurite outgrowth. (A) Ingenuity Pathway Analysis identifies the small molecule pharmacological compound, Panobinostat, predicted to promote CITED2 expression. **(B-C)** Neurite outgrowth (TUJ, grey) following vehicle or 50 nM, 100 nM, or 200 nM Panobinostat in cultured DRG neurons. One-way ANOVA with Tukey's post hoc test; *n*=3 biological replicates. Treatment: f(3,8)=13.29, p<0.01. Scale bar: 200μm. **(D-E)** CITED2 expression (green) 1 after delivery of vehicle or 200 nM Panobinostat in DRG cultured neurons (TUJ, magenta) (Vehicle: 1010±50.93; Panobinostat: 1250±114.2, p<0.05 by t-test, *n=3* biological replicates). Scale bar: 50μm. **(F)** Relative fold change in Cited2 mRNA expression level normalized to beta-actin, assessed by qPCR analysis from cultured DRGs 2 hours after vehicle or 200 nM Panobinostat delivery (Vehicle: 1.0±0.00; Panobinostat: 1.86±0.62, p<0.05 by t-test, *n=3* biological replicates). **(G-H)** Mean neurite outgrowth of cultured DRG neurons electroporated with CRISPR-Cas9-GFP double nickase vs double nickase plasmid containing a non-targeting guide RNA and GFP followed by delivery of vehicle or 200 nM Panobinostat. One-way ANOVA with Tukey's post hoc test; *n*=3 biological replicates. Treatment: f(3,8)=30.02, p<0.001. Scale bar: 50 μm. **(I-J)** Representative micrographs of acCBP/EP300 expression (green) after delivery of vehicle or 200 nM Panobinostat in DRG cultured neurons (TUJ, magenta) (Vehicle: 2418±352.0; Panobinostat: 6329±428.2, p<0.001 by t-test, *n=3* biological replicates). Scale bar: 50μm. **(K-L)** H3k9ac (green) 1

after delivery of vehicle or 200 nM Panobinostat in DRG cultured neurons (TUJ, magenta) (Vehicle: 1396±177.5; Panobinostat: 3489±262.4, $p<0.001$ by t-test, *n=3* biological replicates). Scale bar: 50 µm. **(M-N)** H3k27ac (green) after delivery of vehicle or 200 nM Panobinostat in DRG cultured neurons (TUJ, magenta) (Vehicle: 1290±141.5; Panobinostat: 2792±119.5, $p<0.0001$ by t-test, *n=3* biological replicates). Scale bar: 50 µm.

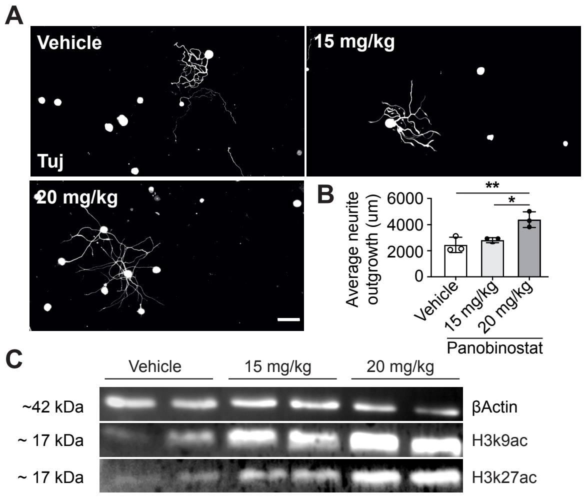

Appendix Figure S7. *Ex vivo* delivery of 20 mg/kg Panobinostat promotes neurite outgrowth and histone H3 acetylation. (A-B) *Ex vivo* neurite outgrowth (TuUJ, grey).Vehicle, 15 mg/kg, or 20 mg/kg Panobinostat delivered systemically via i.p. injections for five days. On day five, the last injection is given two hours before mice are killed and DRG dissected and processed for cell culture. DRG neurons are cultured for 24 hours prior to neurite outgrowth analysis. One-way ANOVA with Tukey's post hoc test; *n*=3 biological replicates. Treatment: $f(2,6)=12.81$, $p<0.01$. **(C)** Western blot analysis of H3k9ac and H3k27ac from sciatic DRG following daily treatment with vehicle or Panobinostat for five days. On day five, the last injection is given two hours before mice are killed and DRG dissected and processed for Western blotting. Band intensity has been normalized against beta-actin. Scale bar: 100 µm.

Table EV1: Predicted protein interactors of CITED2

Name	FP Class score	Name	FP Class score
**UBE2I**	0.8826	**NPM1**	0.5376
**EP300**	0.8826	**JMY**	0.5371
**CREBBP**	0.8826	**THRA**	0.5357
**JUN**	0.8695	**RORA**	0.5352
**TFAP2A**	0.8695	**SMAD2**	0.5325
**BCL6**	0.8354	**SREBF2**	0.5196
**MAF**	0.7793	**NCOA6**	0.5165
**PROX1**	0.7659	**DERA**	0.5123
**GATA4**	0.7532	**RUNX1**	0.5112
**NFIL3**	0.727	**STAT6**	0.5096
**HNF4A**	0.7167	**YBX1**	0.5075
**FOXO1**	0.7158	**MED14**	0.5028
**CTNNB1**	0.7113	**NAP1L1**	0.4962
**SP1**	0.6885	**NFATC2**	0.4949
**TP53**	0.6798	**PCNA (PCNA)**	0.4949
**NR0B2**	0.673	**EGR1**	0.4949
**VDR**	0.6562	**TAL1**	0.4847
**MAP3K5**	0.6529	**CDX2**	0.4826
**FOS**	0.6428	**PTMA**	0.4824
**SS18**	0.6384	**BCL3**	0.4813
**AR**	0.6358	**CITED4**	0.4801
**E2F5**	0.6287	**ELK1**	0.4776
**NFYB**	0.6287	**YY1**	0.4747
**STAT3**	0.6264	**DDX5**	0.4723
**KLF5**	0.6181	**GPS2**	0.4679
**ETS2**	0.6181	**MYOD1**	0.4645
**CEBPB**	0.6146	**RUNX3**	0.4565
**TCF3**	0.6143	**NFKBIA**	0.4556
**NBN**	0.6104	**KLF6**	0.4533
**TFAP2C**	0.5956	**SOX9**	0.4484
**HIF1A**	0.5936	**DTX1**	0.4456
**MEF2A**	0.5914	**STAT2**	0.441
**CREB1**	0.5864	**GTF2B**	0.438
**APC**	0.5856	**STAT5B**	0.4356
**ZNHIT3**	0.5812	**HIST4H4**	0.4323
**SMAD4**	0.5749	**MEF2D**	0.4304
**SMAD7**	0.5731	**ZBTB17**	0.4284
**MAML1**	0.5655	**HNRNPU**	0.4224
**TFAP2B**	0.5644	**MEF2C**	0.4217
**SMAD3**	0.5617	**ATF4**	0.4201
**PAX6**	0.5596	**RB1**	0.4198
**TWIST1**	0.5561	**MED1**	0.4148

**NR2C2**	0.5549	**RECQL4**	0.4136
**PPARG**	0.5548	**STAT5A**	0.4136
**HMGN2**	0.5509	**TP73**	0.4124
**ETV4**	0.5439	**FHL2**	0.4099
**IRF2**	0.5416	**IRF3**	0.4087
**ETS1**	0.541	**HNF1A**	0.4078
**PPARD**	0.5401	**TRERF1**	0.406
**HDAC3**	0.54	**MAOA**	0.4011
