# Supplementary figures and images for: CITED2 is a druggable epigenetic switch coupling neuronal maturation to regenerative decline
Source: EMBO Mol Med. 2026 Feb 23;18(4):1174–201. doi: 10.1038/s44321-026-00385-w (PMC13083982; doi:10.1038/s44321-026-00385-w)

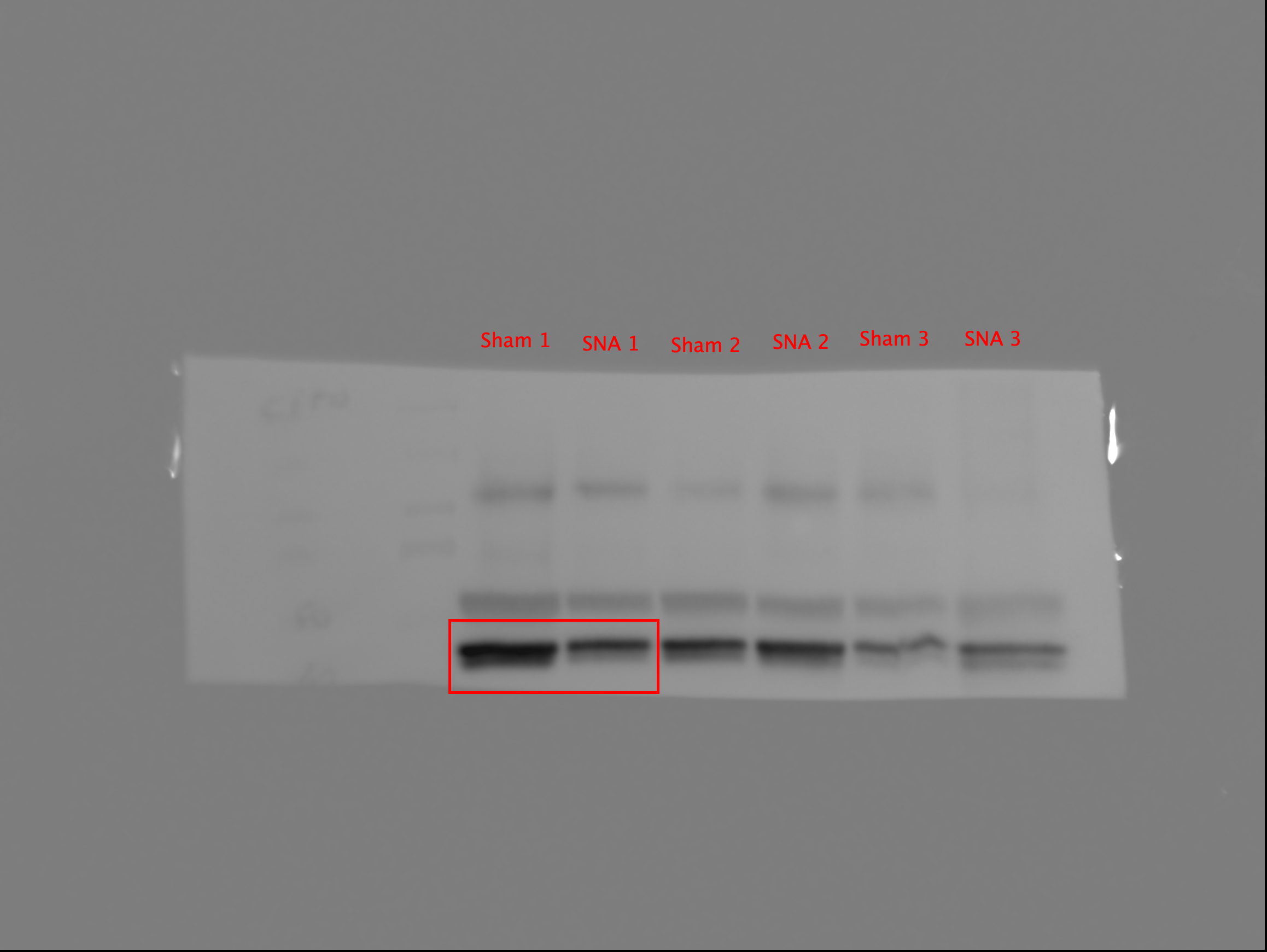

Supplement: Supplementary file 11 — Source data Fig. 2 [file 44321_2026_385_MOESM11_ESM.zip › Source Data_Figure 2/H-I/actin cytoplasmic.tif]

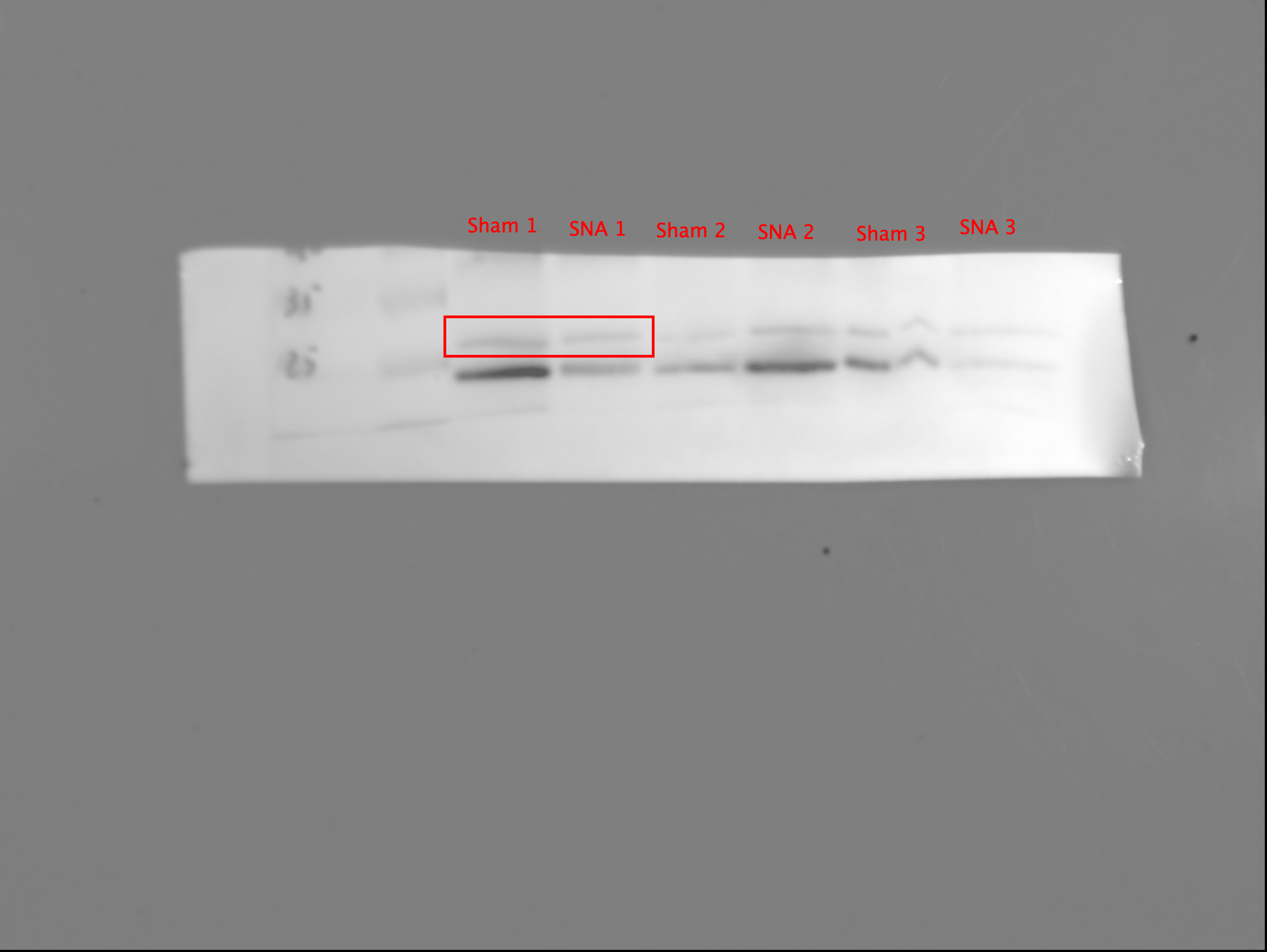

Supplement: Supplementary file 11 — Source data Fig. 2 [file 44321_2026_385_MOESM11_ESM.zip › Source Data_Figure 2/H-I/cited2 cytoplasmic.tif]

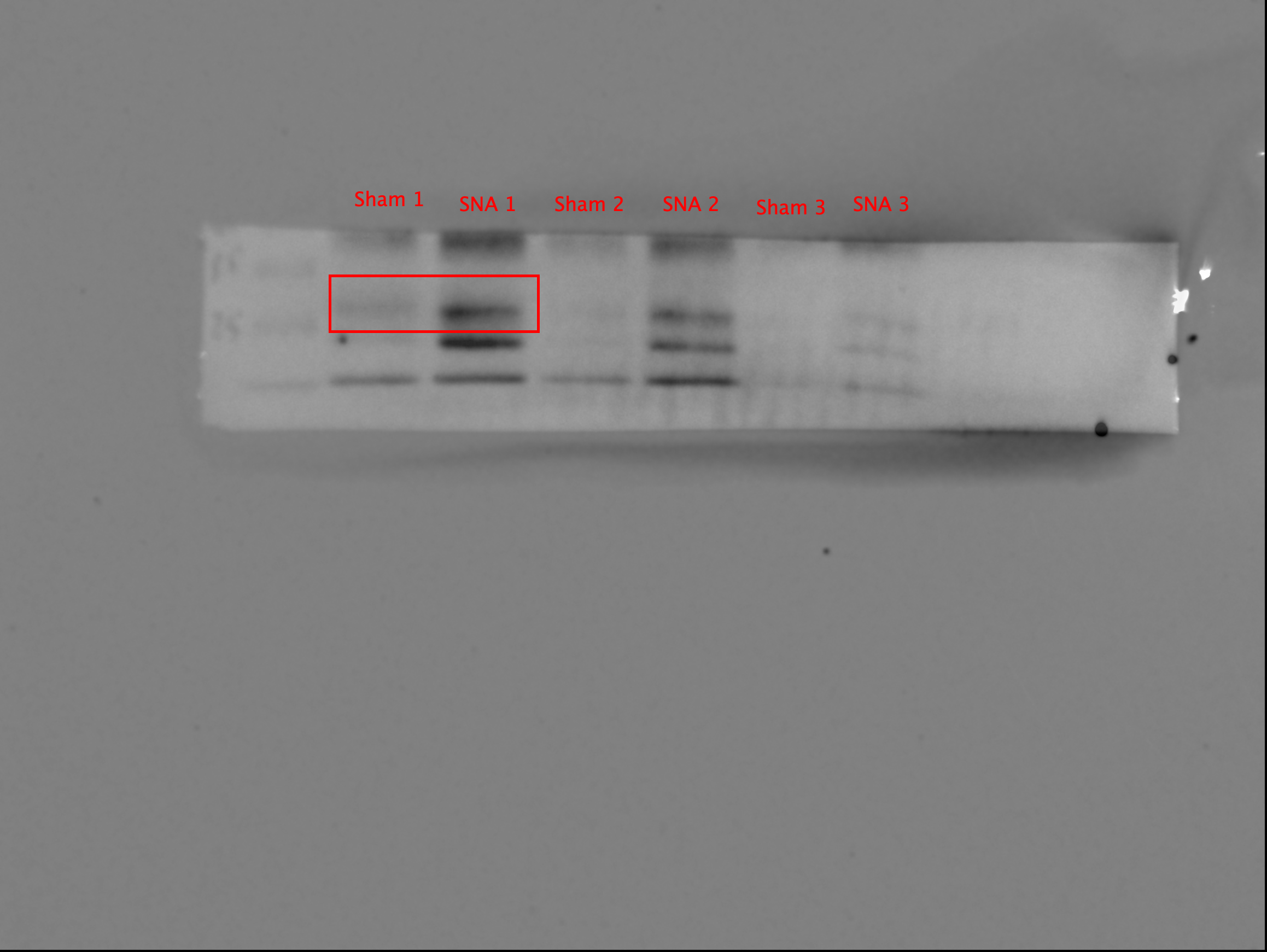

Supplement: Supplementary file 11 — Source data Fig. 2 [file 44321_2026_385_MOESM11_ESM.zip › Source Data_Figure 2/H-I/cited2 nuclear.tif]

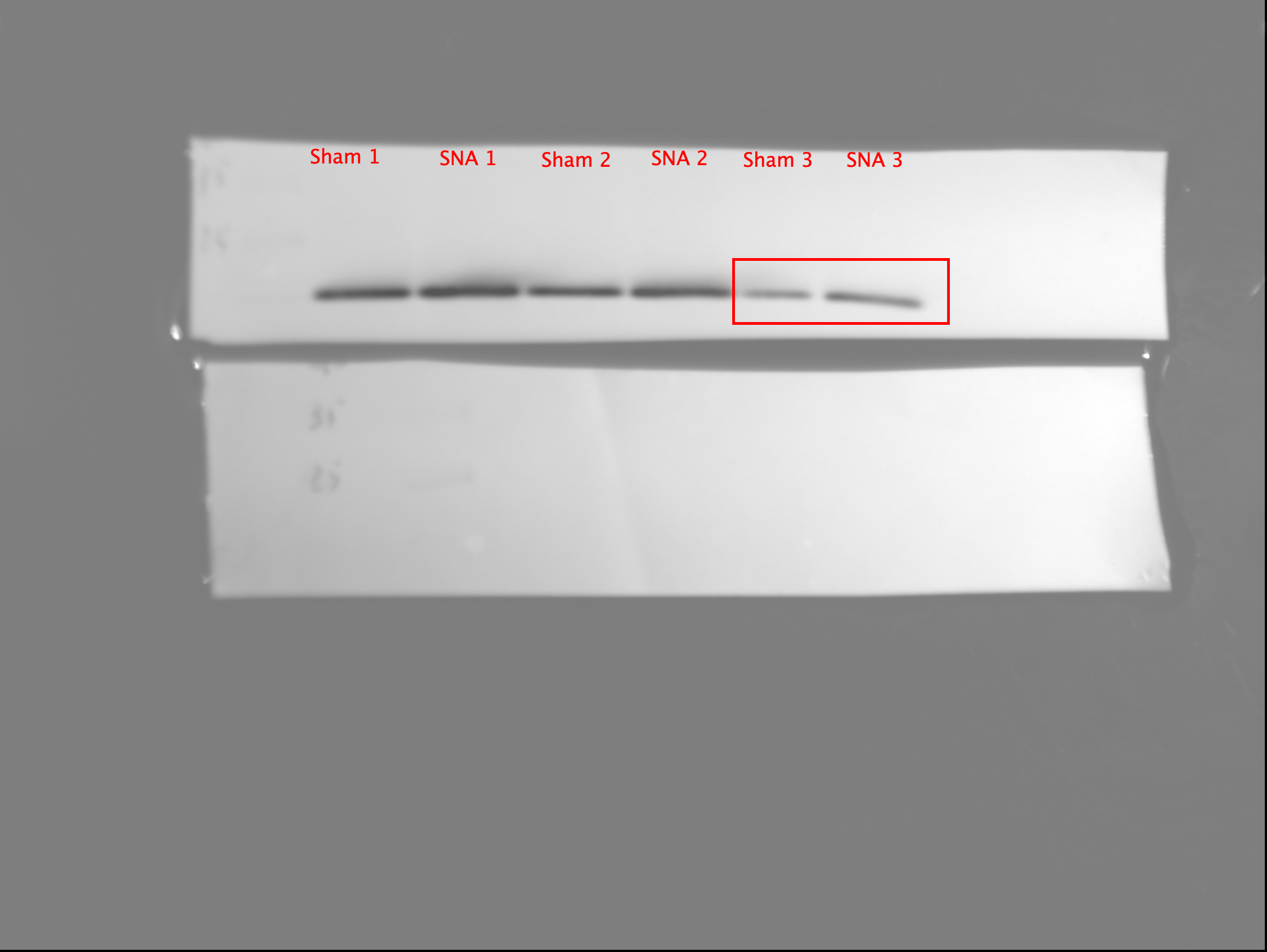

Supplement: Supplementary file 11 — Source data Fig. 2 [file 44321_2026_385_MOESM11_ESM.zip › Source Data_Figure 2/H-I/H3 nuclear.tif]

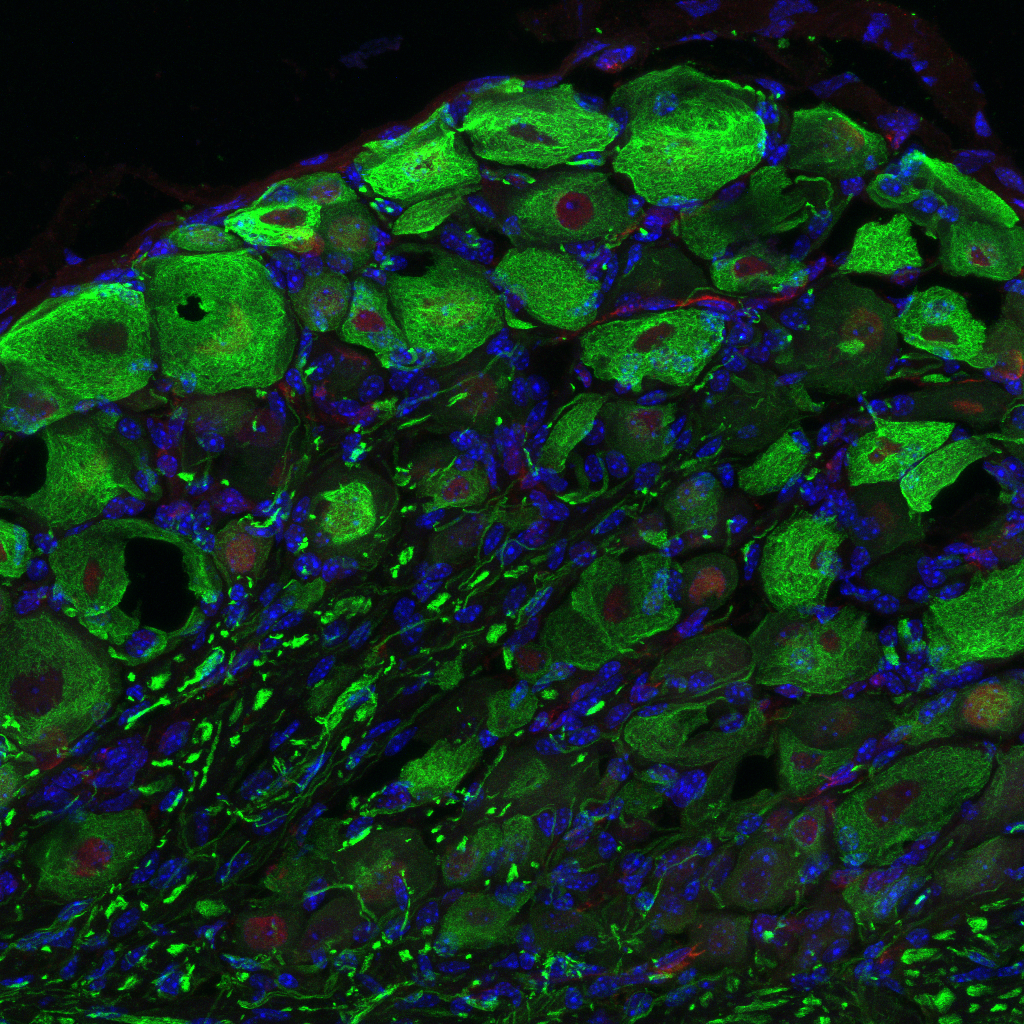

Supplement: Supplementary file 11 — Source data Fig. 2 [file 44321_2026_385_MOESM11_ESM.zip › Source Data_Figure 2/C/endogenous cited2 Sham/endogenous cited2 Sham 40x_001_Processed001.tif]

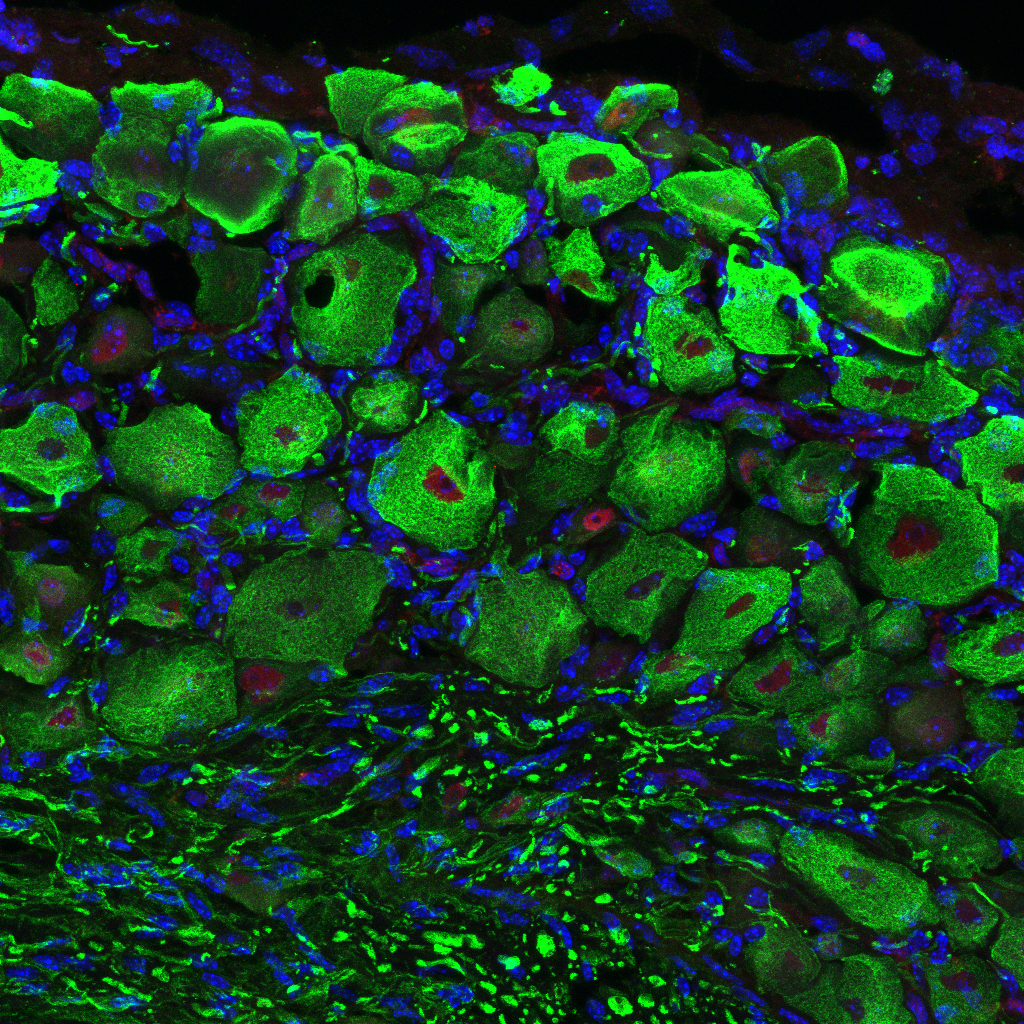

Supplement: Supplementary file 11 — Source data Fig. 2 [file 44321_2026_385_MOESM11_ESM.zip › Source Data_Figure 2/C/endogenous cited2 SNA/endogenous cited2 SNA 40x_001_Processed001.tif]

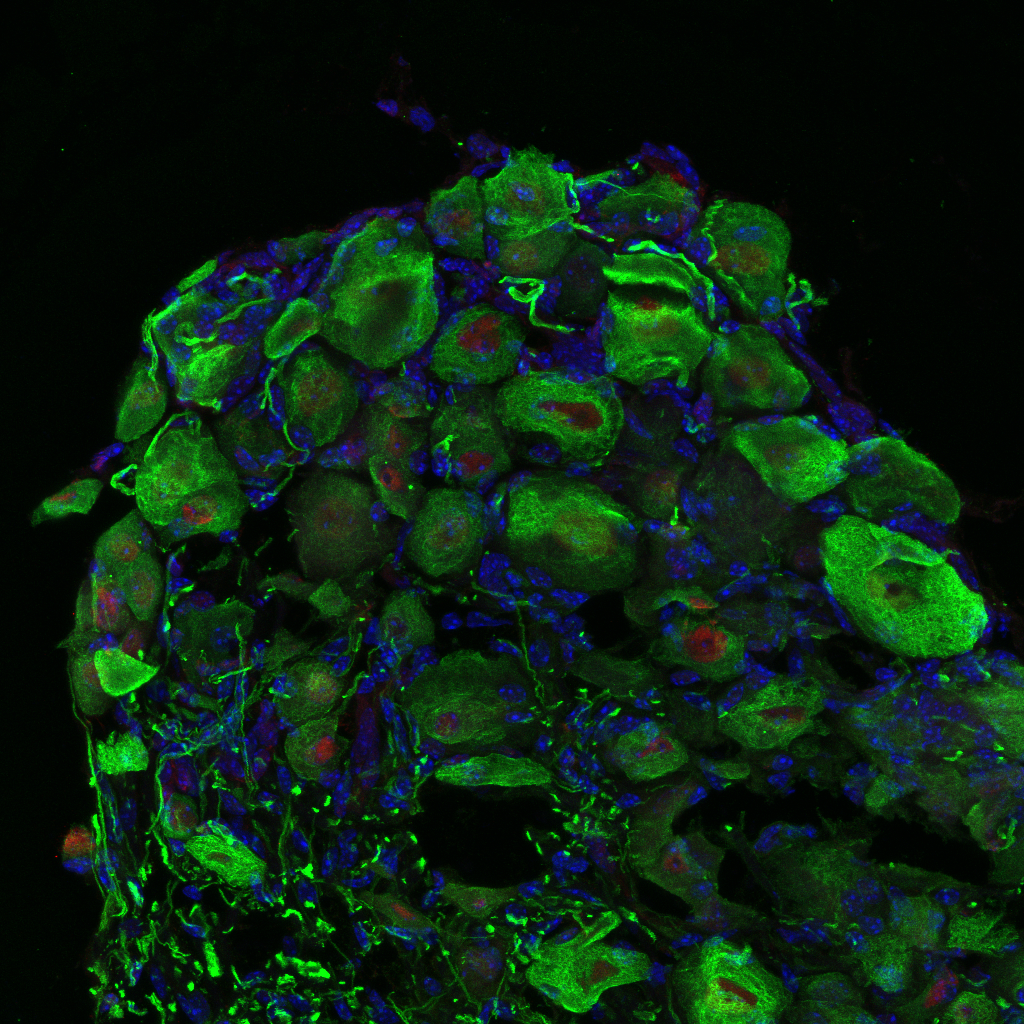

Supplement: Supplementary file 11 — Source data Fig. 2 [file 44321_2026_385_MOESM11_ESM.zip › Source Data_Figure 2/E/endogenous cited2 DCA/endogenous cited2 DCA 40x_001_Processed001.tif]

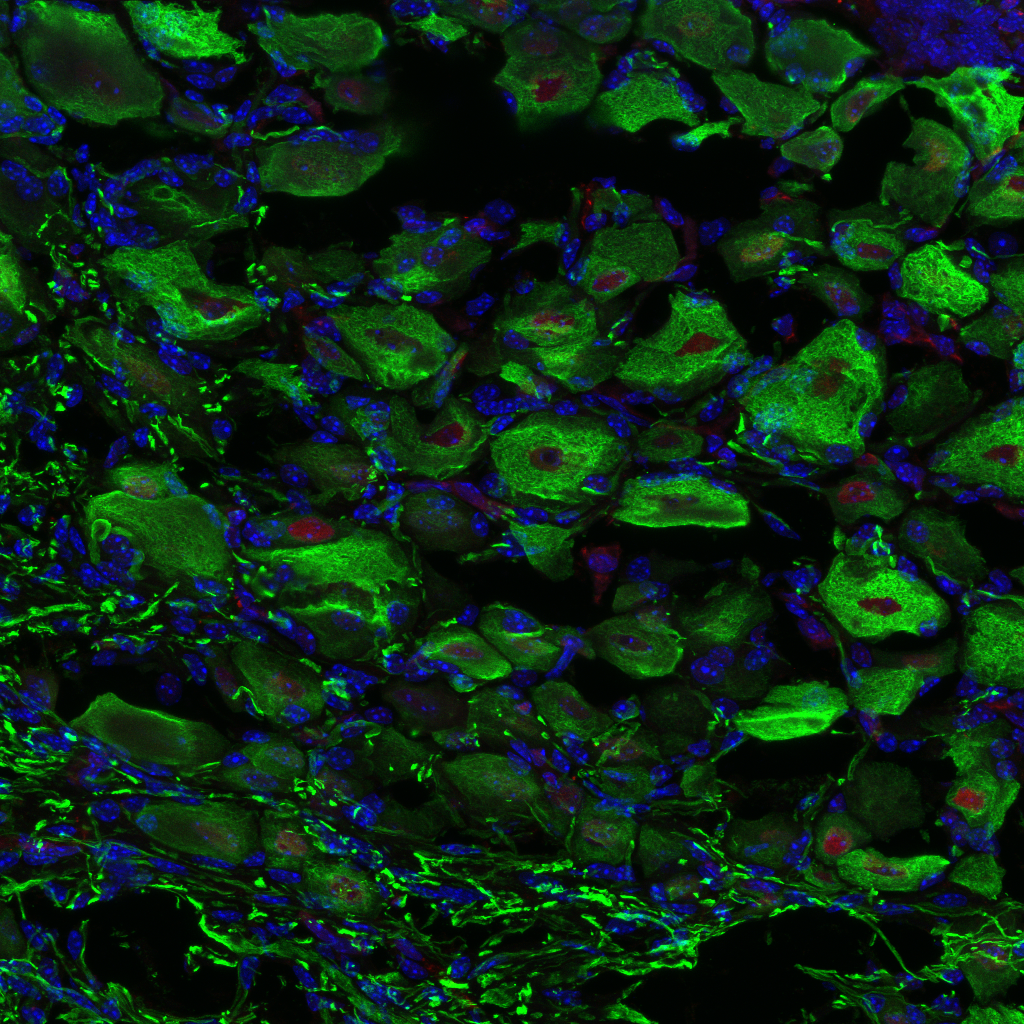

Supplement: Supplementary file 11 — Source data Fig. 2 [file 44321_2026_385_MOESM11_ESM.zip › Source Data_Figure 2/E/endogenous cited2 LAM/endogenous cited2 LAM 40x_001_Processed001.tif]

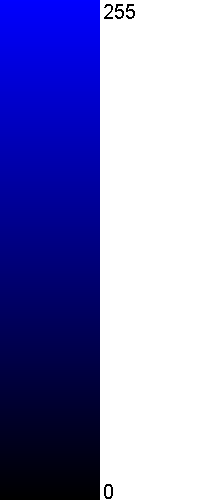

Supplement: Supplementary file 11 — Source data Fig. 2 [file 44321_2026_385_MOESM11_ESM.zip › Source Data_Figure 2/E/endogenous cited2 DCA/MetaData/endogenous cited2 DCA 40x_001_Processed001ch0LUT.png]

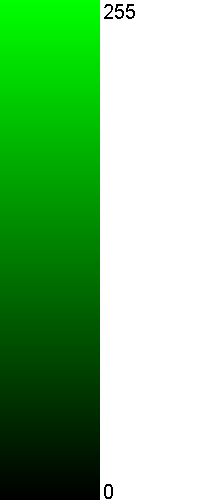

Supplement: Supplementary file 11 — Source data Fig. 2 [file 44321_2026_385_MOESM11_ESM.zip › Source Data_Figure 2/E/endogenous cited2 DCA/MetaData/endogenous cited2 DCA 40x_001_Processed001ch1LUT.png]

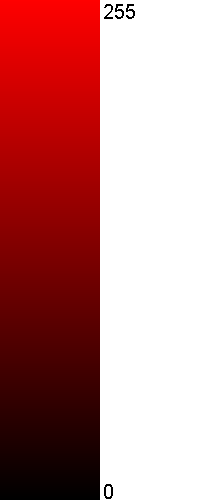

Supplement: Supplementary file 11 — Source data Fig. 2 [file 44321_2026_385_MOESM11_ESM.zip › Source Data_Figure 2/E/endogenous cited2 DCA/MetaData/endogenous cited2 DCA 40x_001_Processed001ch2LUT.png]

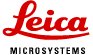

Supplement: Supplementary file 11 — Source data Fig. 2 [file 44321_2026_385_MOESM11_ESM.zip › Source Data_Figure 2/E/endogenous cited2 DCA/MetaData/LeicaLogo.jpg]

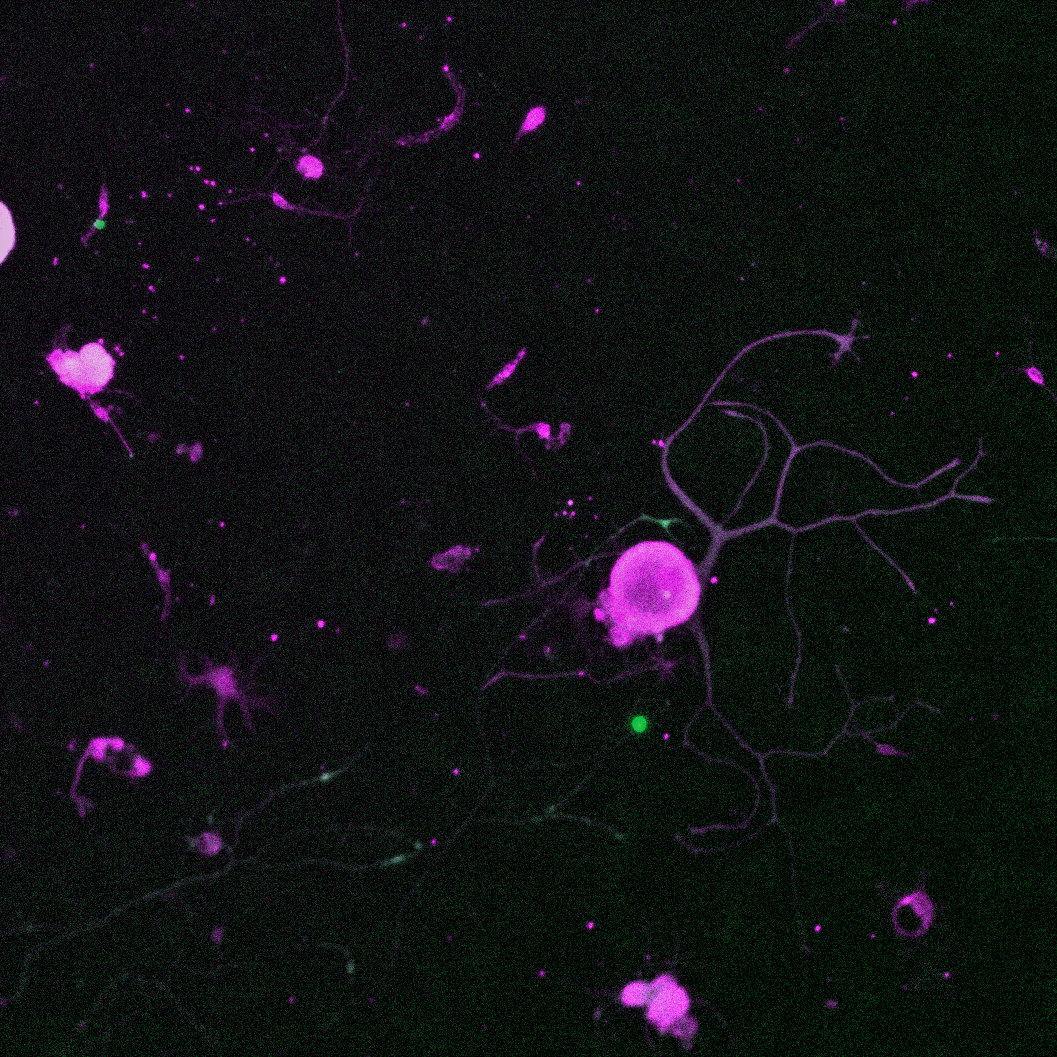

Supplement: Supplementary file 12 — Source data Fig. 3 [file 44321_2026_385_MOESM12_ESM.zip › Source Data_Figure 3/A/Cited2KD neurite outgrowth.tif]

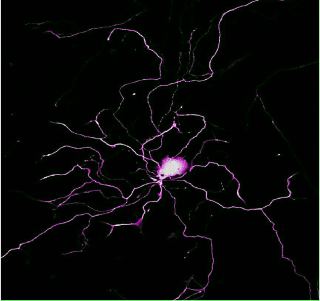

Supplement: Supplementary file 12 — Source data Fig. 3 [file 44321_2026_385_MOESM12_ESM.zip › Source Data_Figure 3/A/GFP neurite outgrowth.tif]

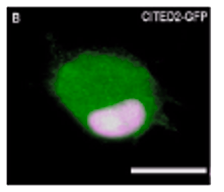

Supplement: Supplementary file 12 — Source data Fig. 3 [file 44321_2026_385_MOESM12_ESM.zip › Source Data_Figure 3/C/C2_DRGculture_plasmid.tif]

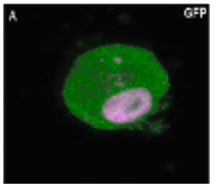

Supplement: Supplementary file 12 — Source data Fig. 3 [file 44321_2026_385_MOESM12_ESM.zip › Source Data_Figure 3/C/GFP_DRGculture_plasmid.tif]

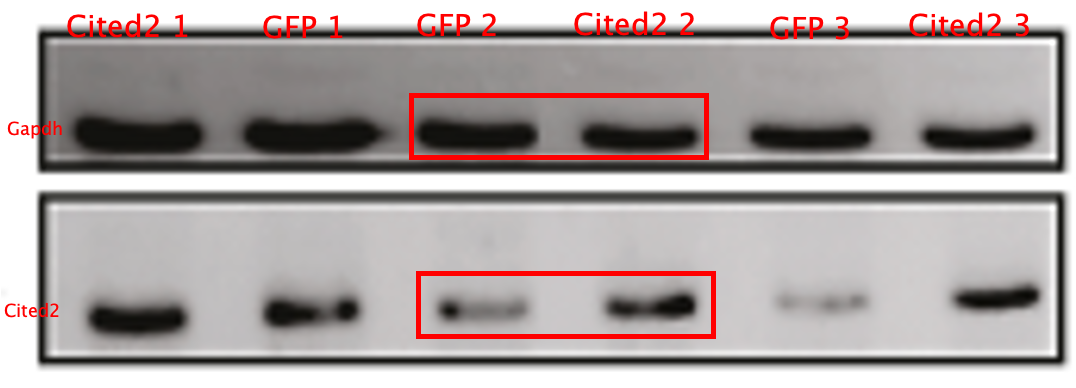

Supplement: Supplementary file 12 — Source data Fig. 3 [file 44321_2026_385_MOESM12_ESM.zip › Source Data_Figure 3/E/Cited2vGFP_WB.tif]

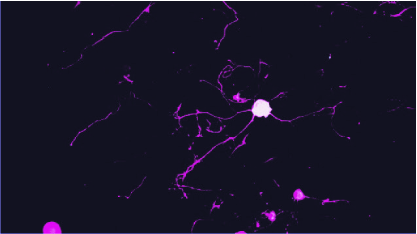

Supplement: Supplementary file 12 — Source data Fig. 3 [file 44321_2026_385_MOESM12_ESM.zip › Source Data_Figure 3/G-I/myelin C2.tif]

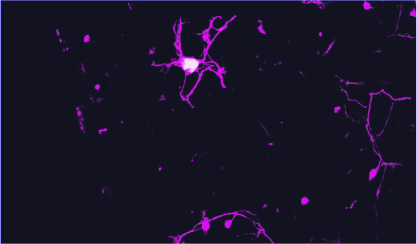

Supplement: Supplementary file 12 — Source data Fig. 3 [file 44321_2026_385_MOESM12_ESM.zip › Source Data_Figure 3/G-I/myelin GFP.tif]

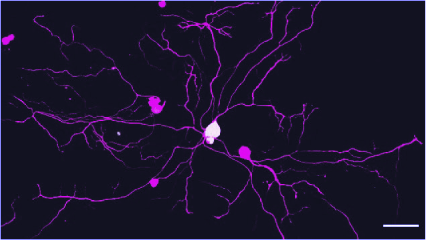

Supplement: Supplementary file 12 — Source data Fig. 3 [file 44321_2026_385_MOESM12_ESM.zip › Source Data_Figure 3/G-I/PDL C2.tif]

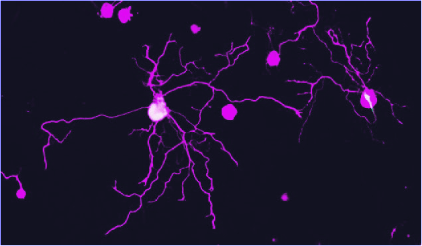

Supplement: Supplementary file 12 — Source data Fig. 3 [file 44321_2026_385_MOESM12_ESM.zip › Source Data_Figure 3/G-I/PDL GFP.tif]

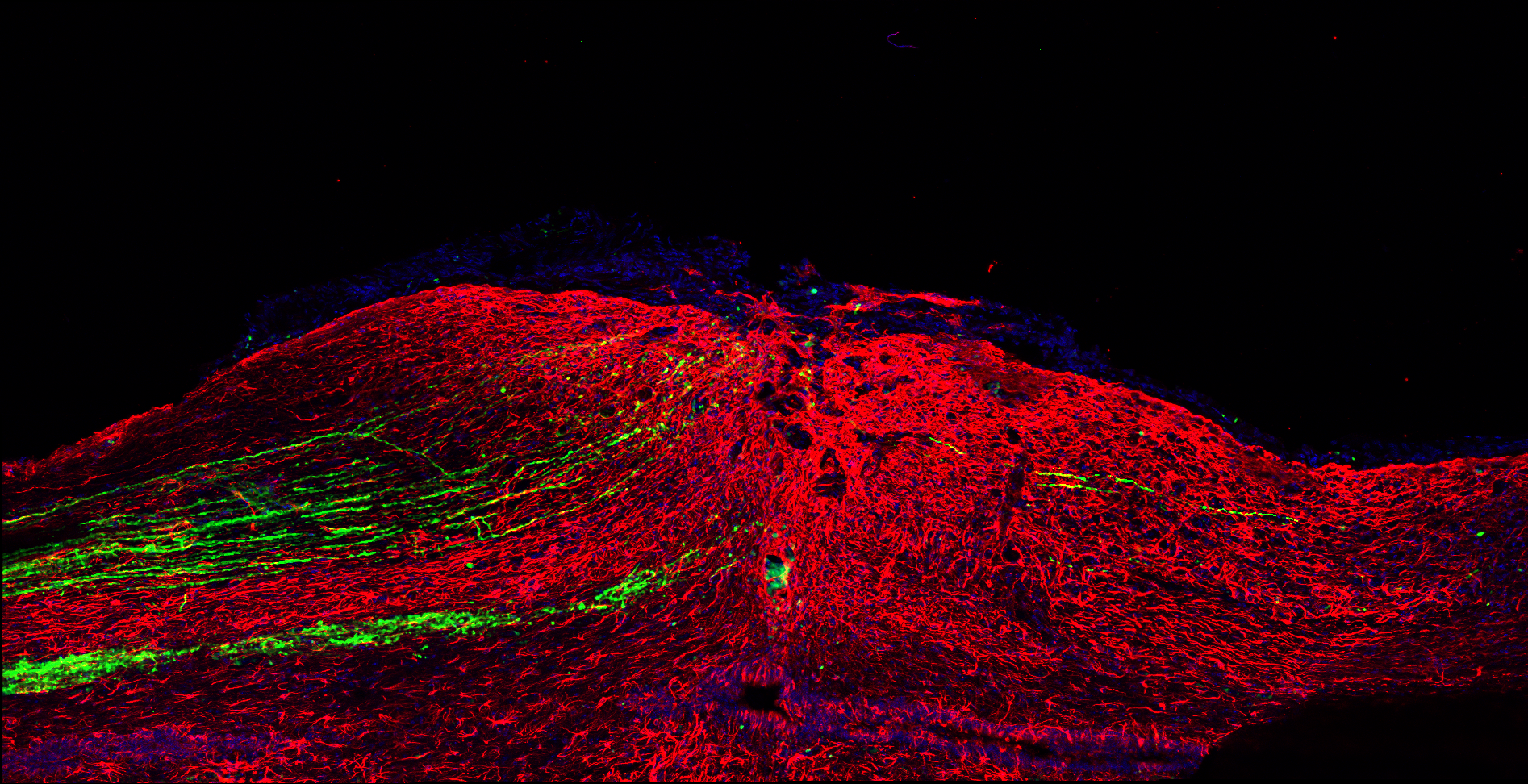

Supplement: Supplementary file 13 — Source data Fig. 4 [file 44321_2026_385_MOESM13_ESM.zip › Source Data_Figure 4/B/Axonal Regen Cited2.tif]

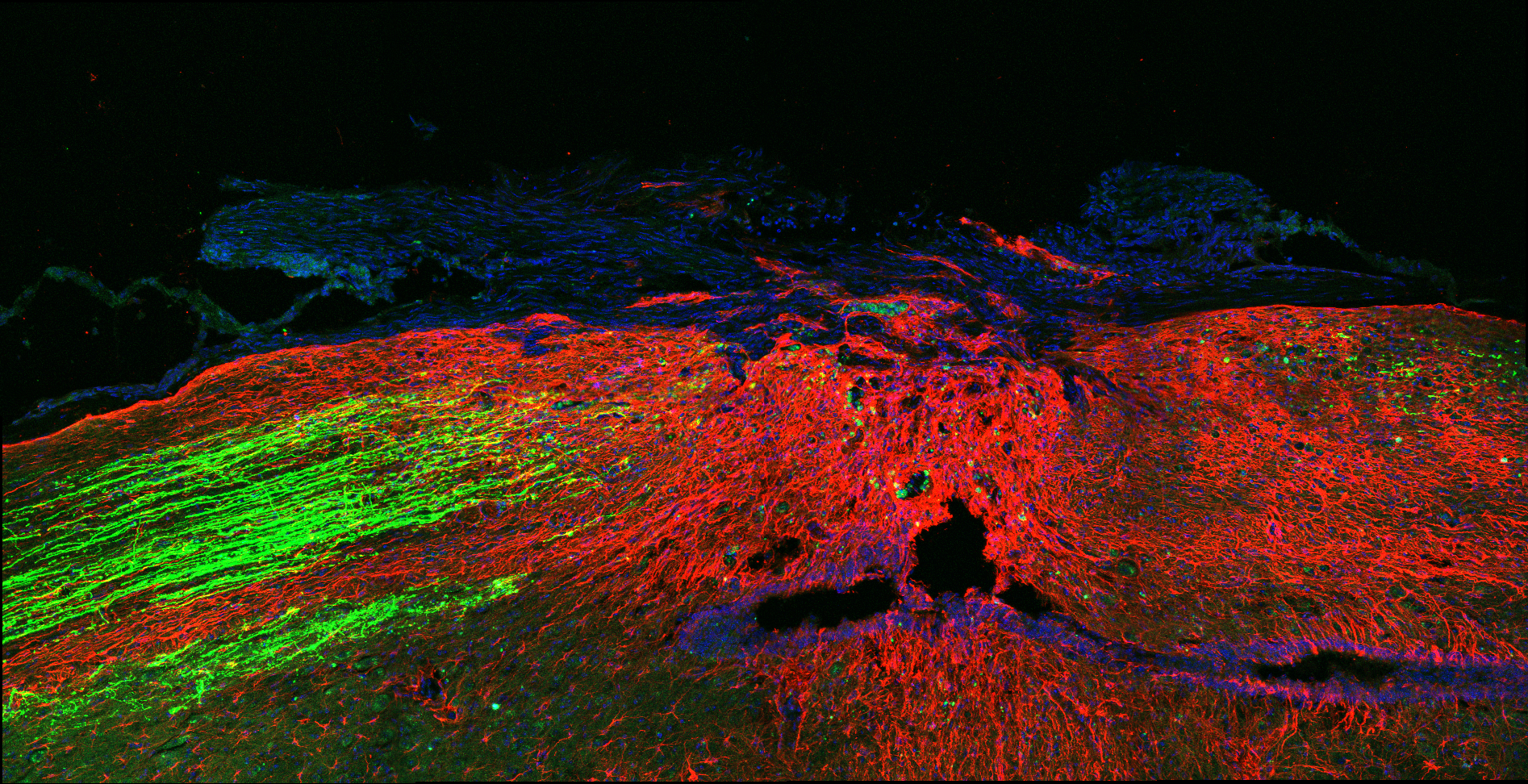

Supplement: Supplementary file 13 — Source data Fig. 4 [file 44321_2026_385_MOESM13_ESM.zip › Source Data_Figure 4/B/Axonal Regen GFP.tif]

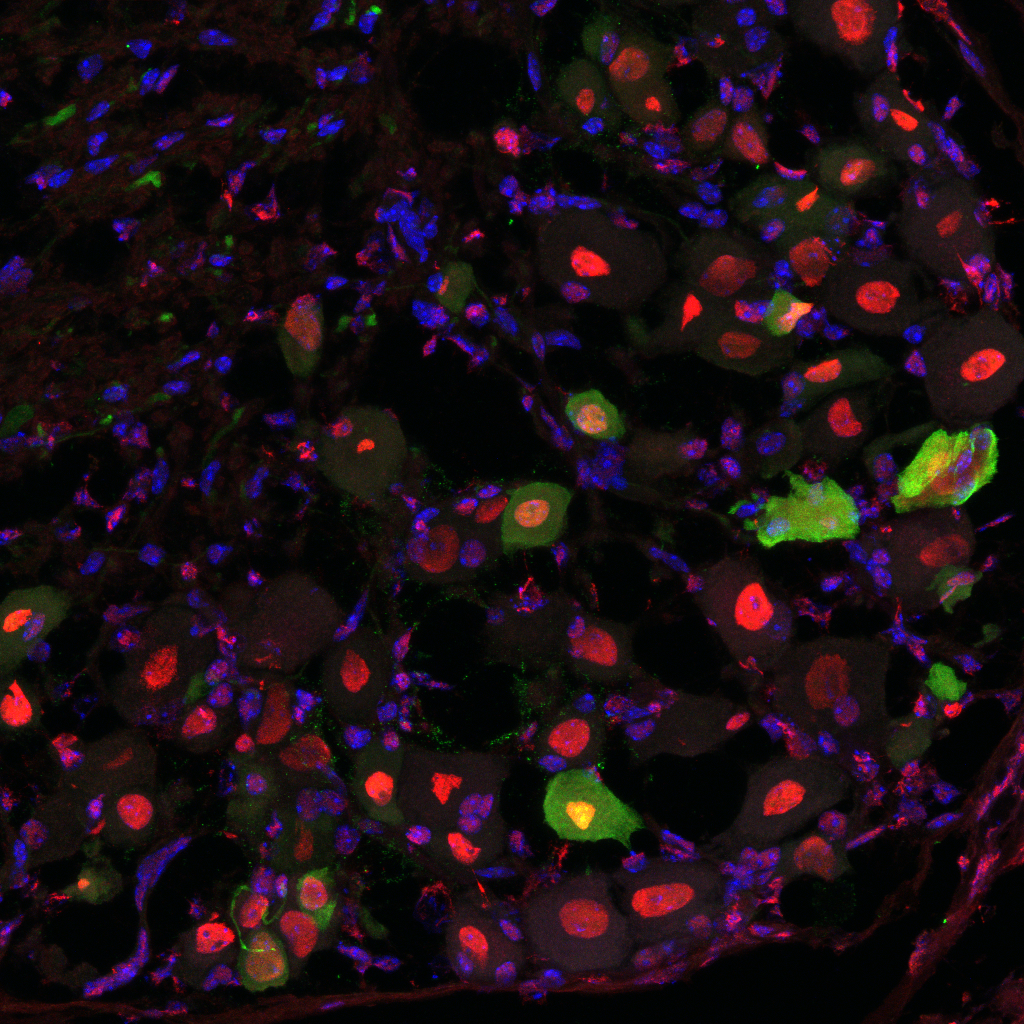

Supplement: Supplementary file 13 — Source data Fig. 4 [file 44321_2026_385_MOESM13_ESM.zip › Source Data_Figure 4/E/Cited2 24HPI.tif]

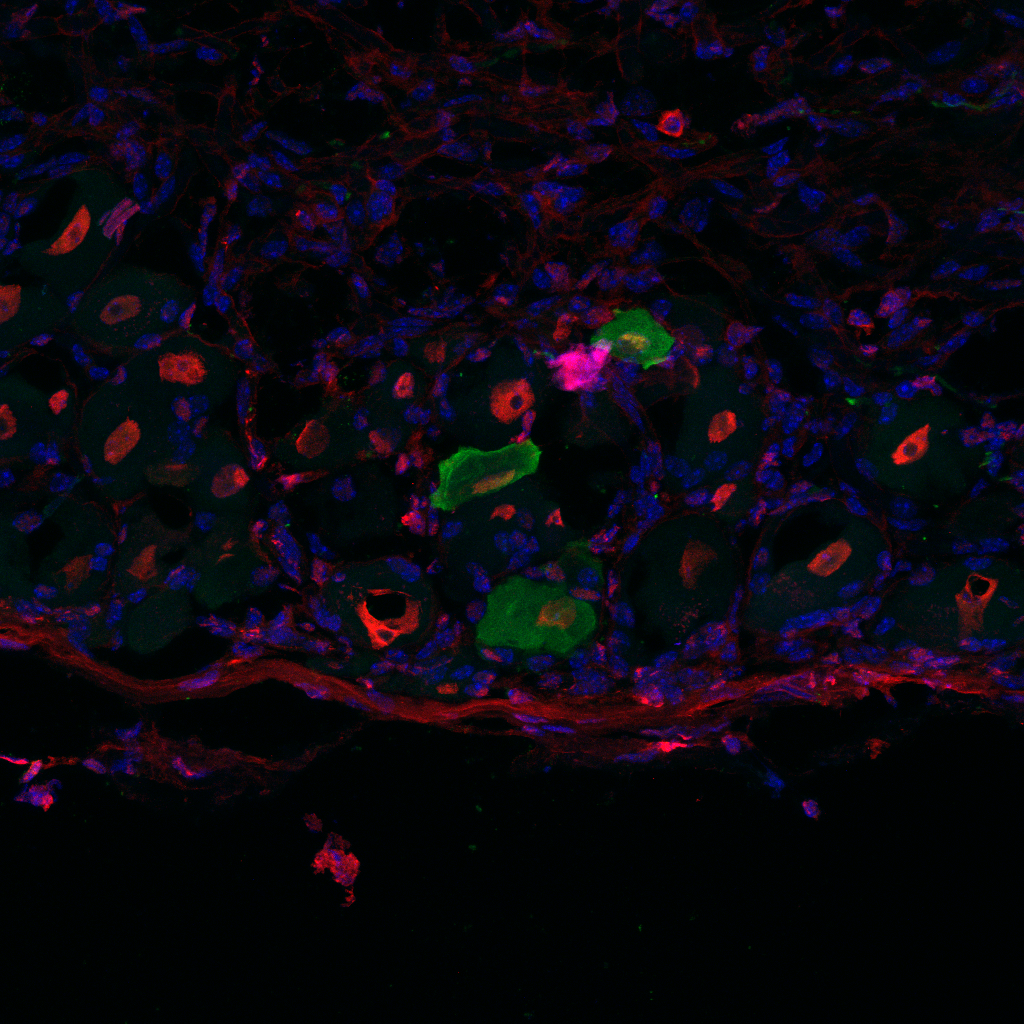

Supplement: Supplementary file 13 — Source data Fig. 4 [file 44321_2026_385_MOESM13_ESM.zip › Source Data_Figure 4/E/GFP 24HPI.tif]

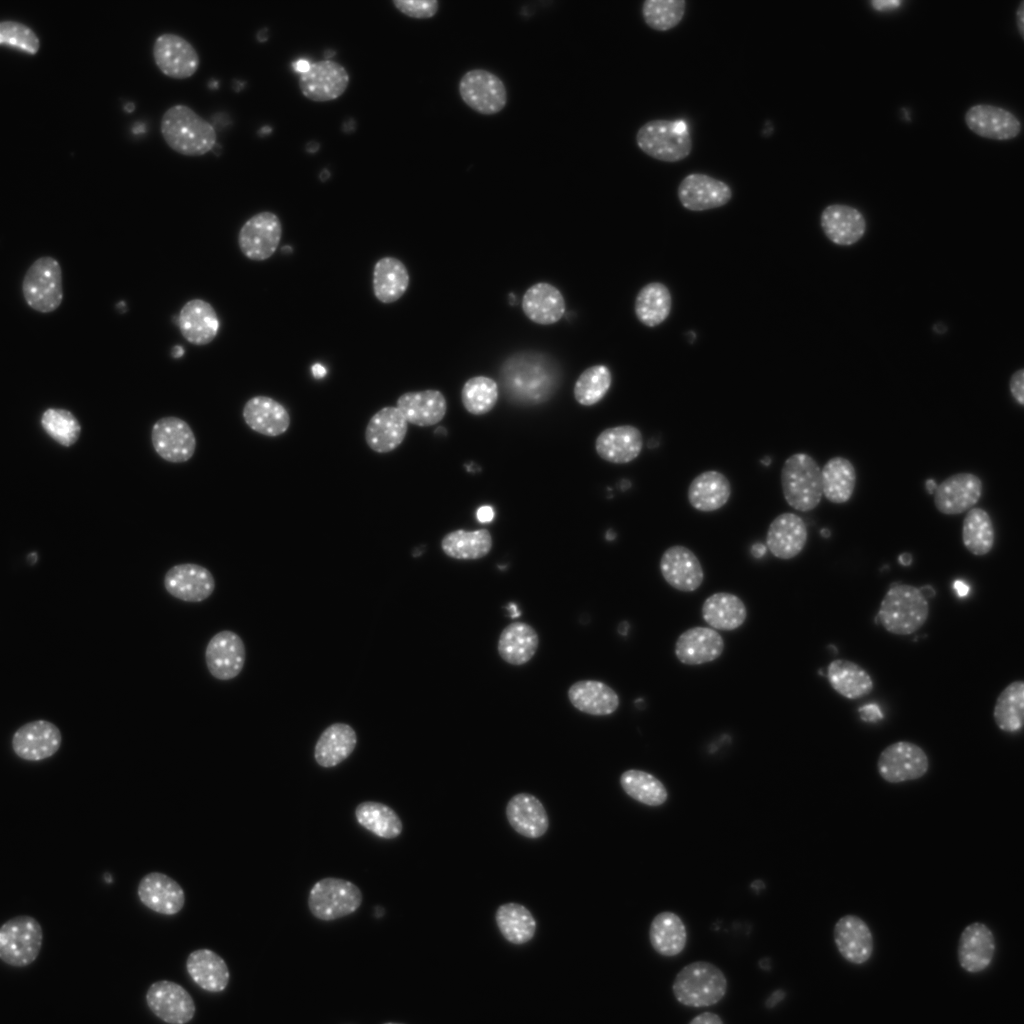

Supplement: Supplementary file 17 — Source data Fig. 8 [file 44321_2026_385_MOESM17_ESM.zip › Source Data_Figure 8/A/Cited2 Bipolar.tif]

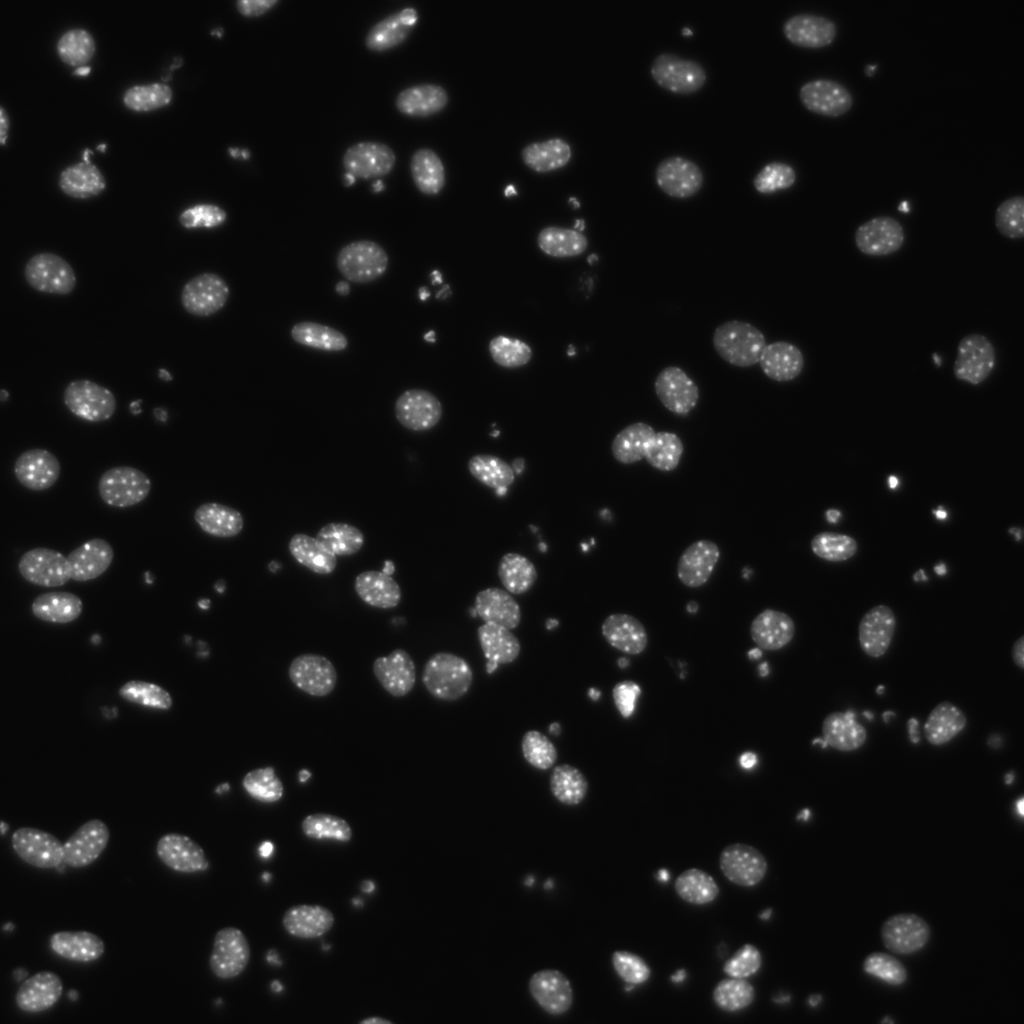

Supplement: Supplementary file 17 — Source data Fig. 8 [file 44321_2026_385_MOESM17_ESM.zip › Source Data_Figure 8/C/Cited2 Cbx5.tif]

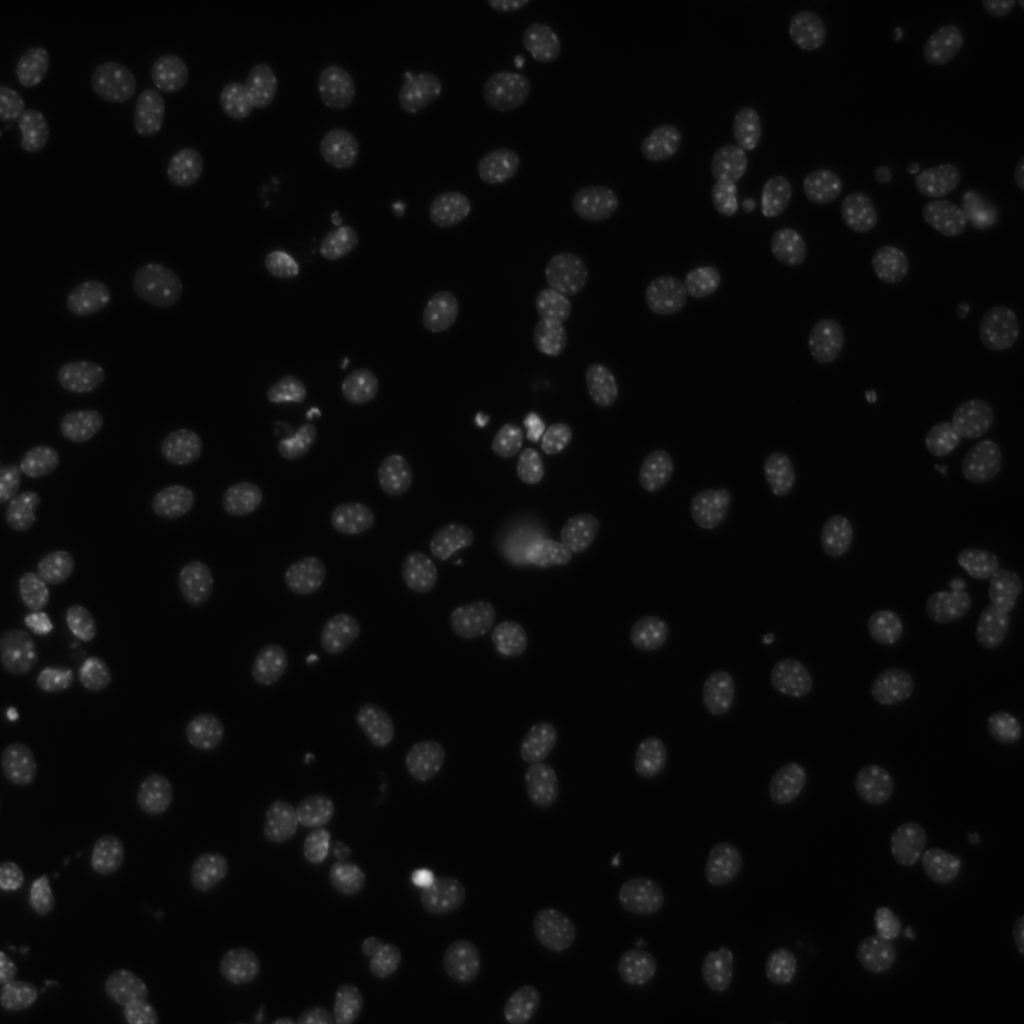

Supplement: Supplementary file 17 — Source data Fig. 8 [file 44321_2026_385_MOESM17_ESM.zip › Source Data_Figure 8/C/GFP Cbx5.tif]

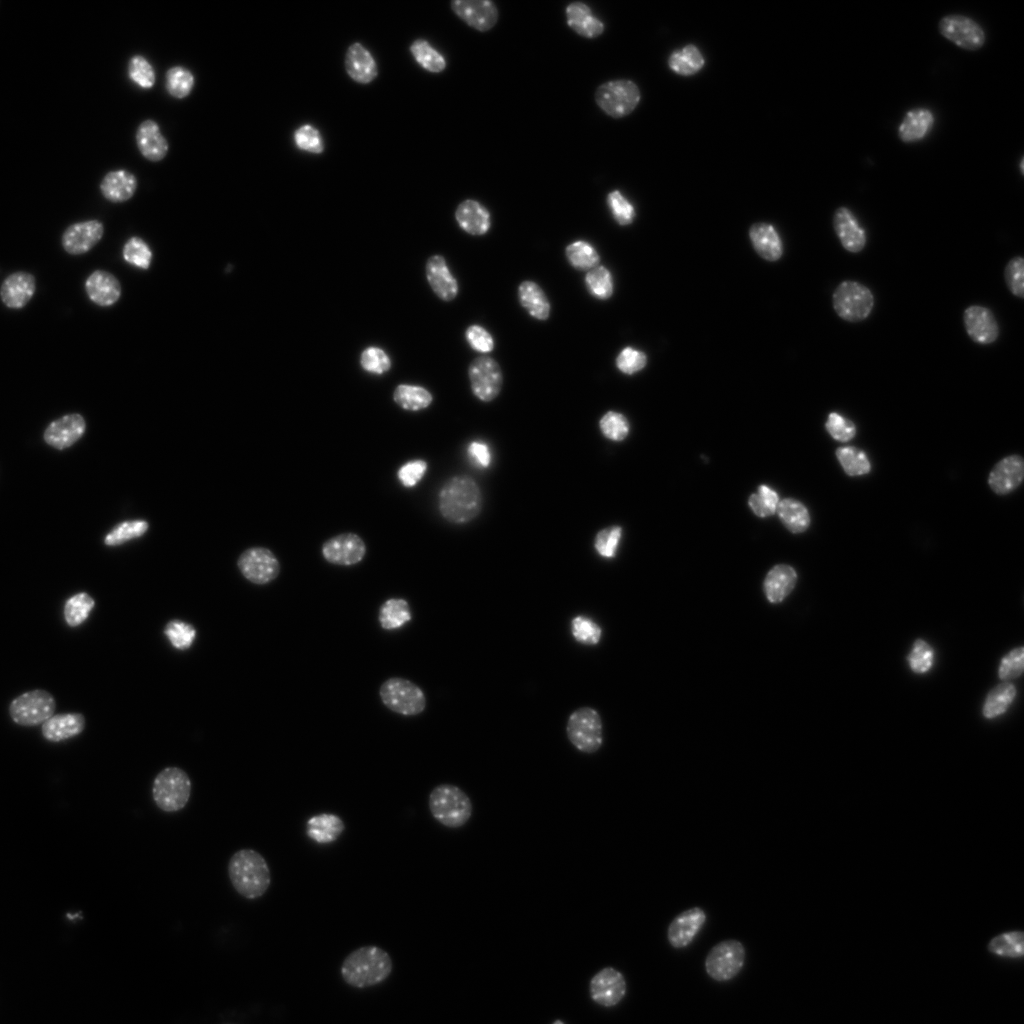

Supplement: Supplementary file 17 — Source data Fig. 8 [file 44321_2026_385_MOESM17_ESM.zip › Source Data_Figure 8/E/Cited2 HoxD3.tif]

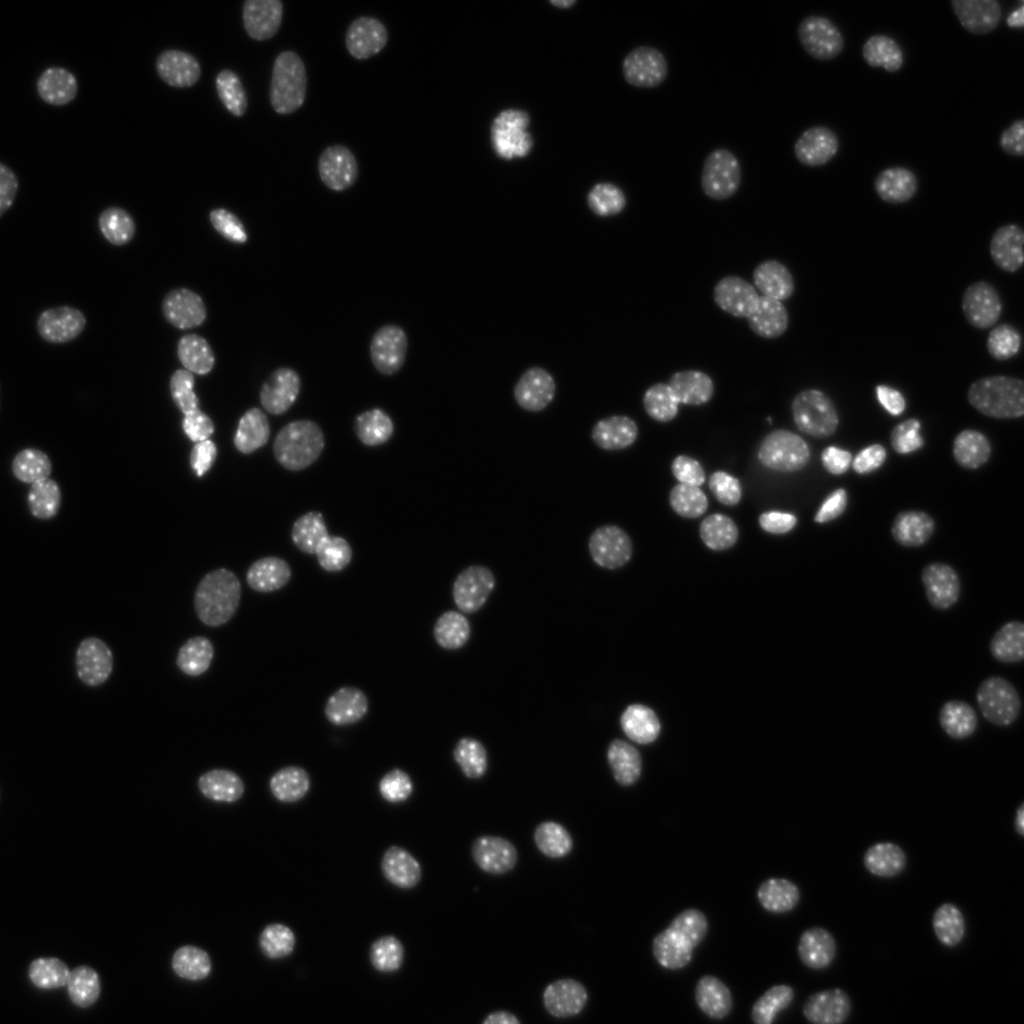

Supplement: Supplementary file 17 — Source data Fig. 8 [file 44321_2026_385_MOESM17_ESM.zip › Source Data_Figure 8/E/GFP HoxD3.tif]

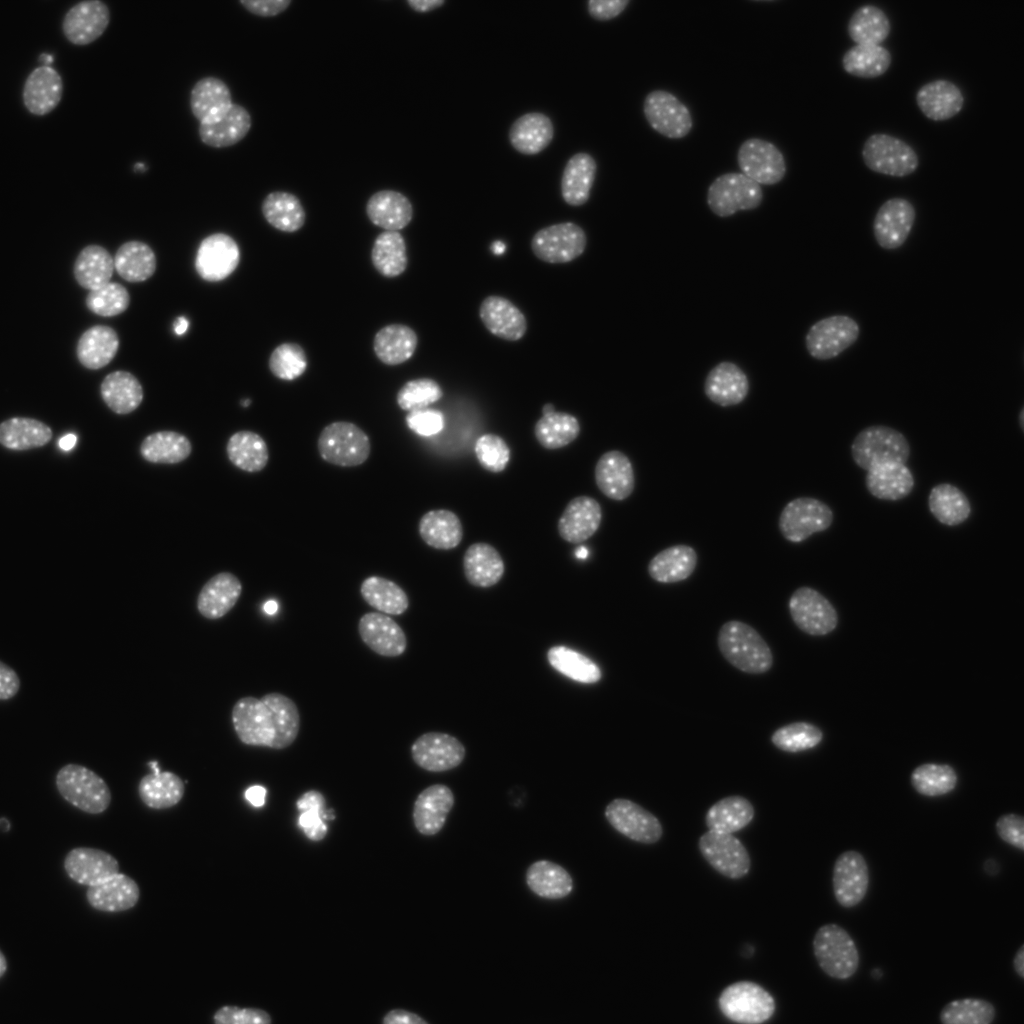

Supplement: Supplementary file 17 — Source data Fig. 8 [file 44321_2026_385_MOESM17_ESM.zip › Source Data_Figure 8/A/Replicates/Cited2 - Bipolar/Bipolar_2.tif]

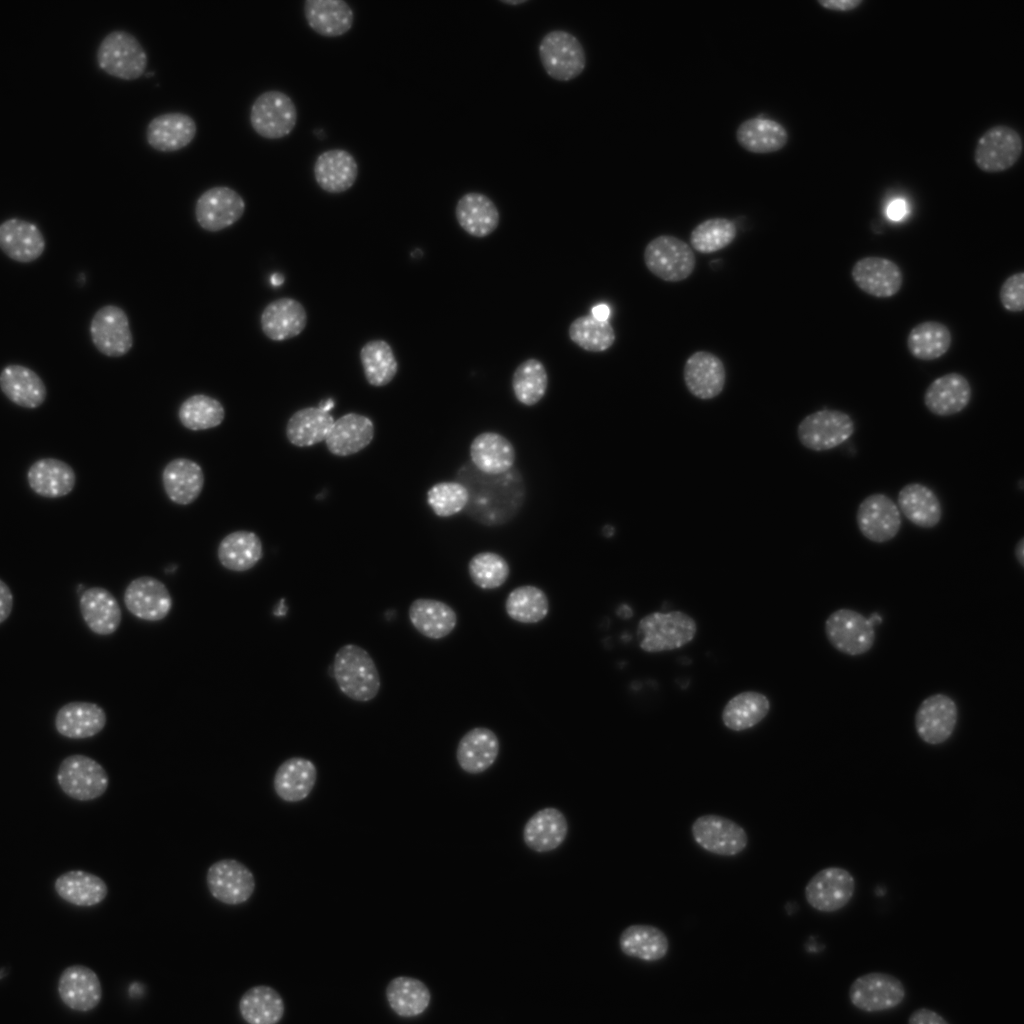

Supplement: Supplementary file 17 — Source data Fig. 8 [file 44321_2026_385_MOESM17_ESM.zip › Source Data_Figure 8/A/Replicates/Cited2 - Bipolar/Bipolar_3.tif]

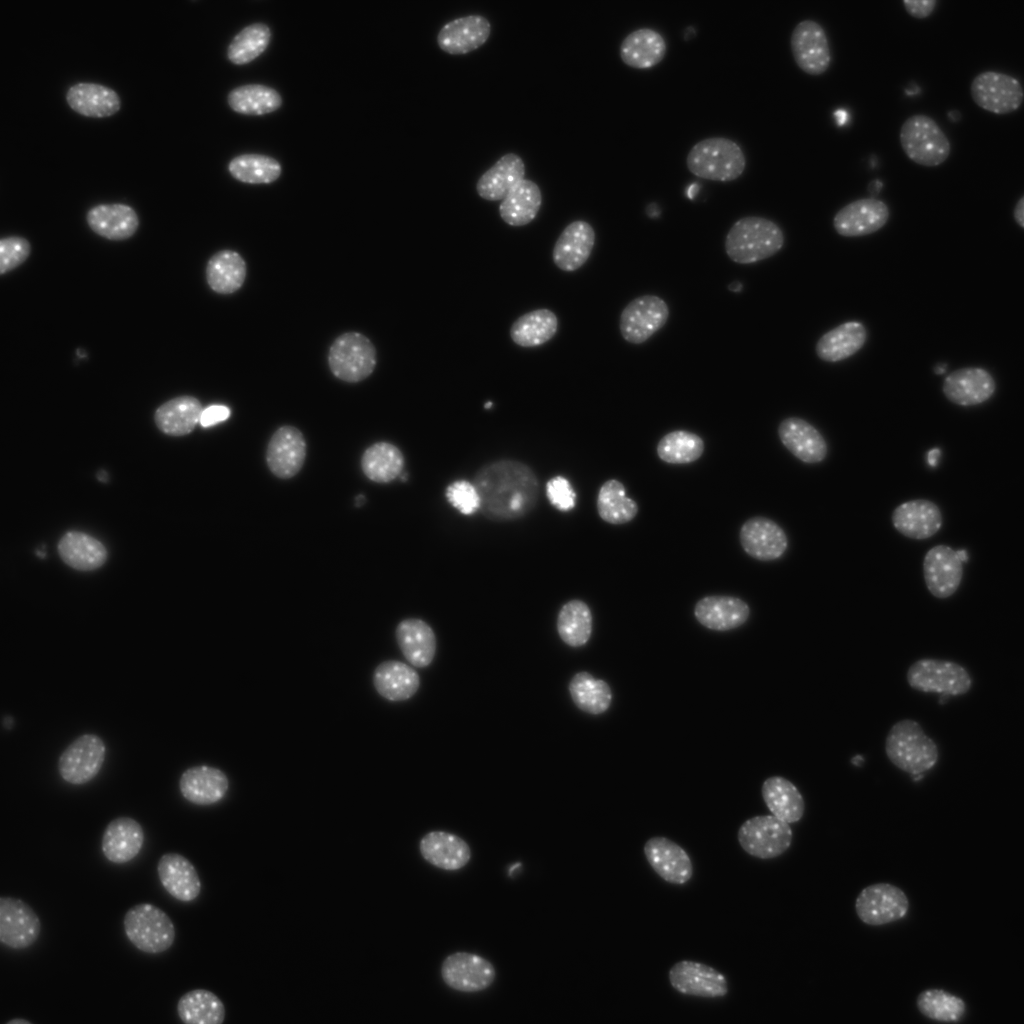

Supplement: Supplementary file 17 — Source data Fig. 8 [file 44321_2026_385_MOESM17_ESM.zip › Source Data_Figure 8/A/Replicates/Cited2 - Multi/Multi_1.tif]

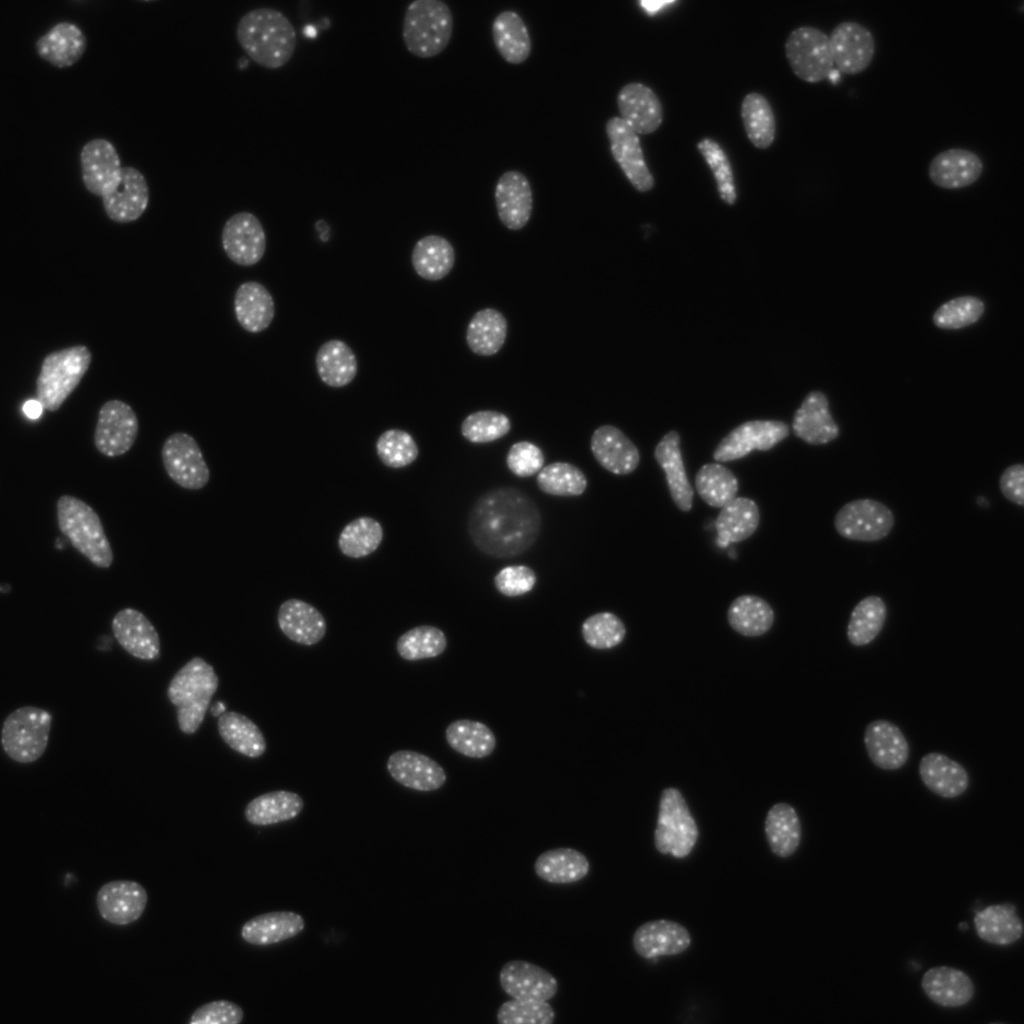

Supplement: Supplementary file 17 — Source data Fig. 8 [file 44321_2026_385_MOESM17_ESM.zip › Source Data_Figure 8/A/Replicates/Cited2 - Multi/Multi_2.tif]

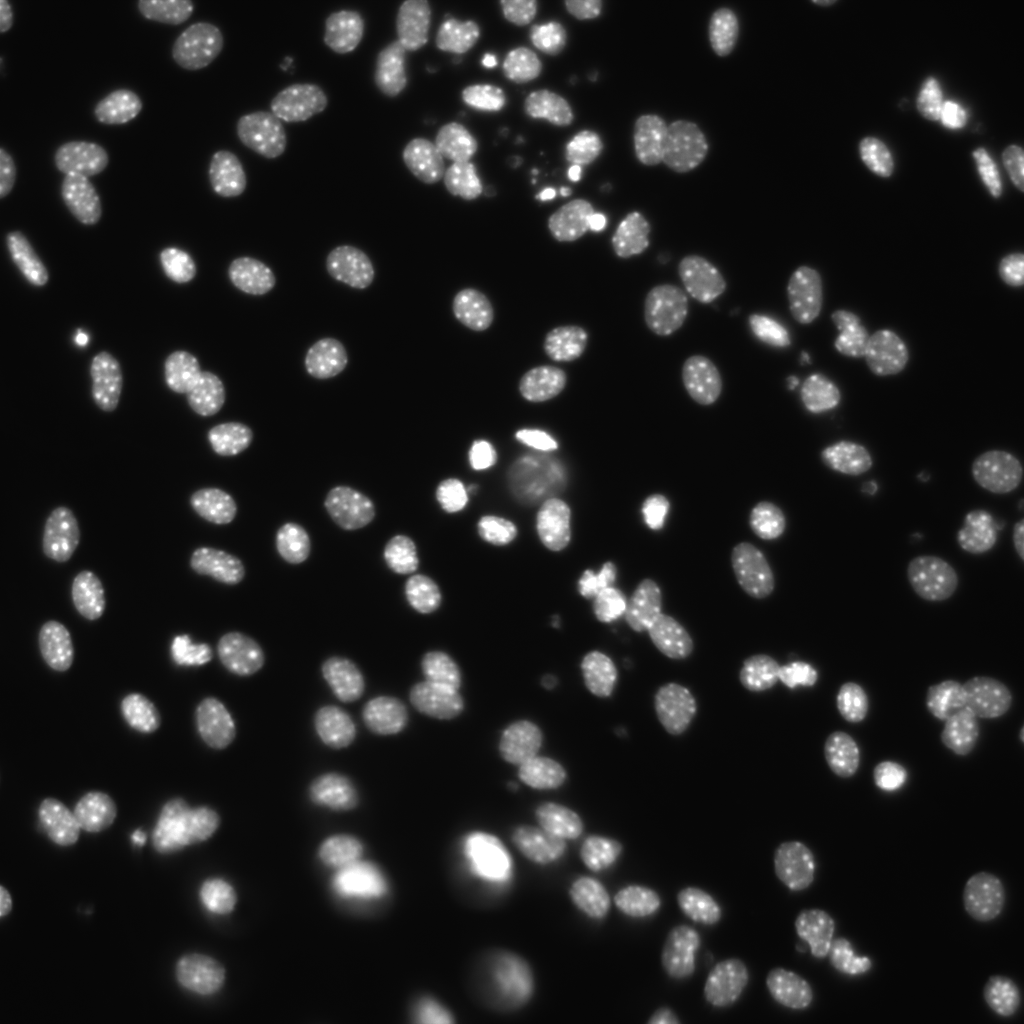

Supplement: Supplementary file 17 — Source data Fig. 8 [file 44321_2026_385_MOESM17_ESM.zip › Source Data_Figure 8/A/Replicates/GFP - Pseudo/Pseudo_1.tif]

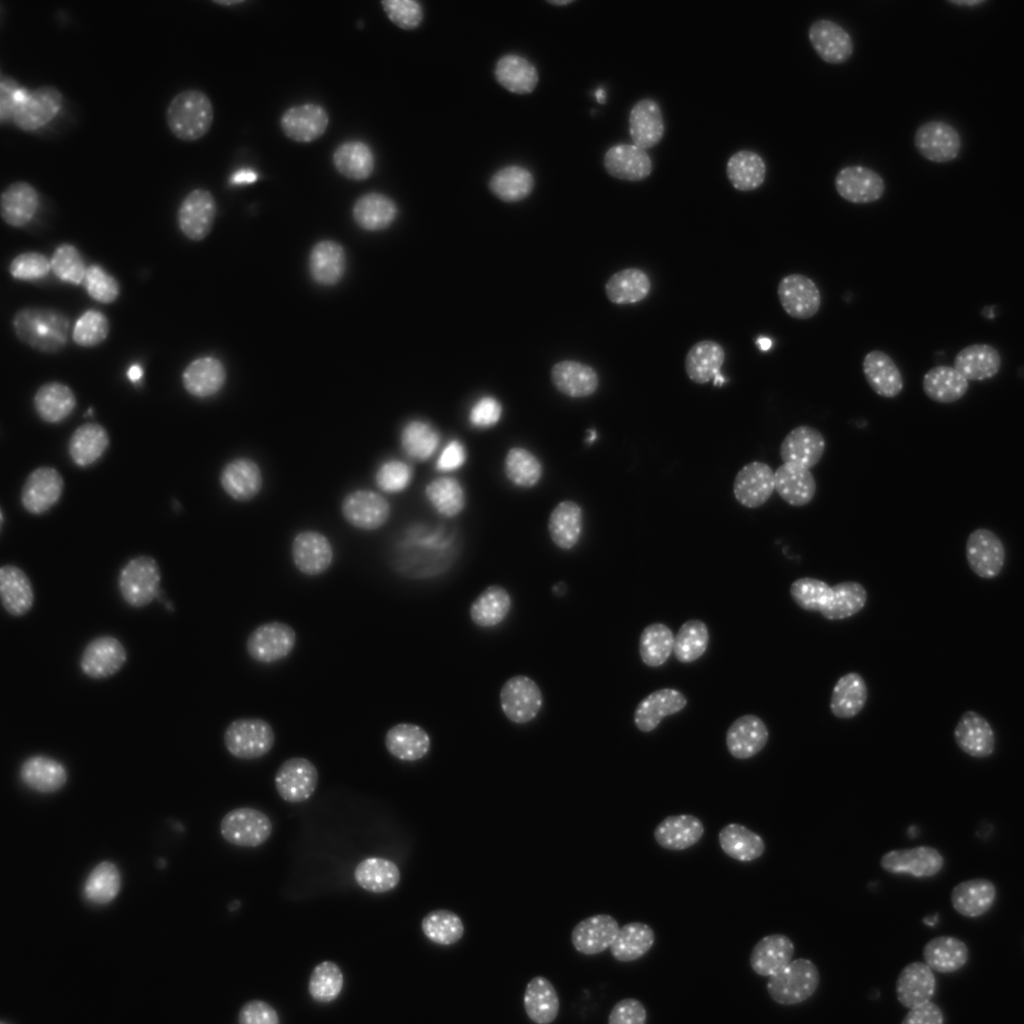

Supplement: Supplementary file 17 — Source data Fig. 8 [file 44321_2026_385_MOESM17_ESM.zip › Source Data_Figure 8/A/Replicates/GFP - Pseudo/Pseudo_2.tif]

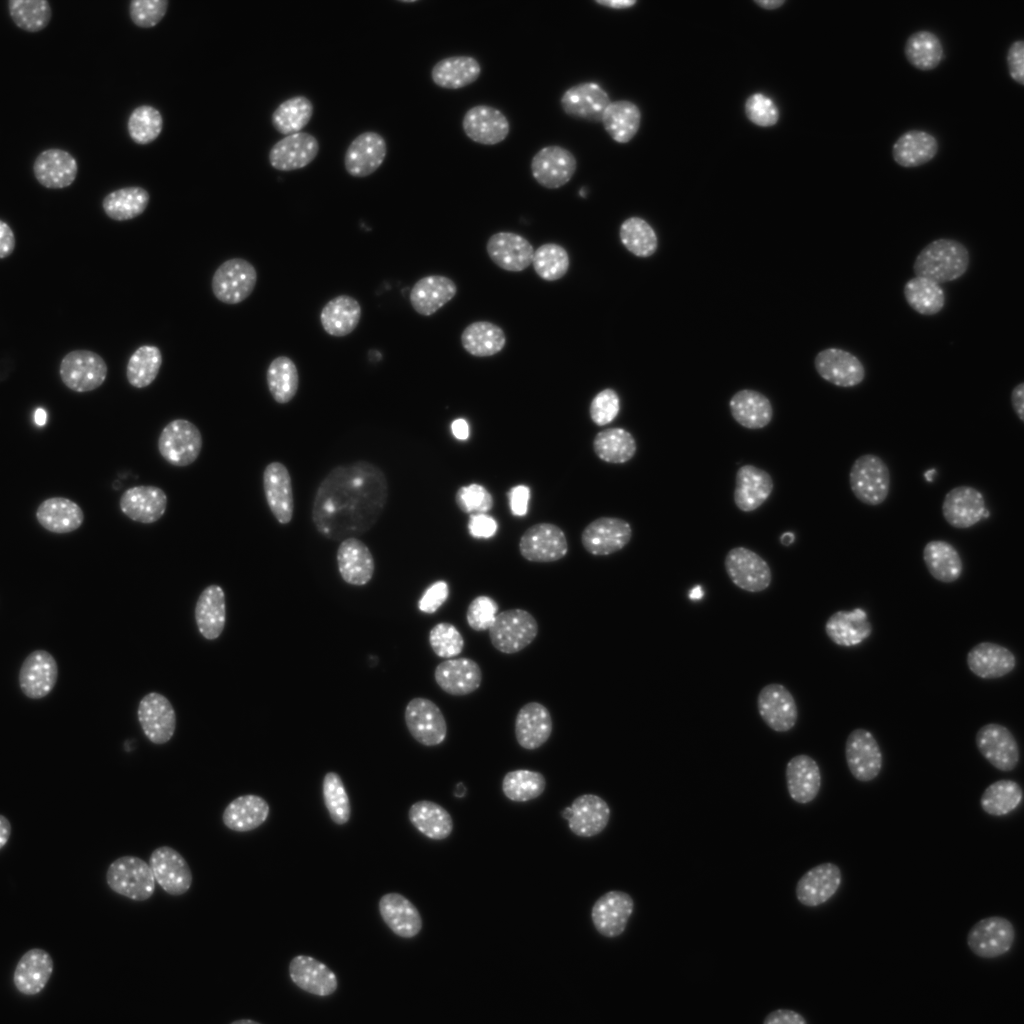

Supplement: Supplementary file 17 — Source data Fig. 8 [file 44321_2026_385_MOESM17_ESM.zip › Source Data_Figure 8/A/Replicates/GFP - Pseudo/Pseudo_3.tif]

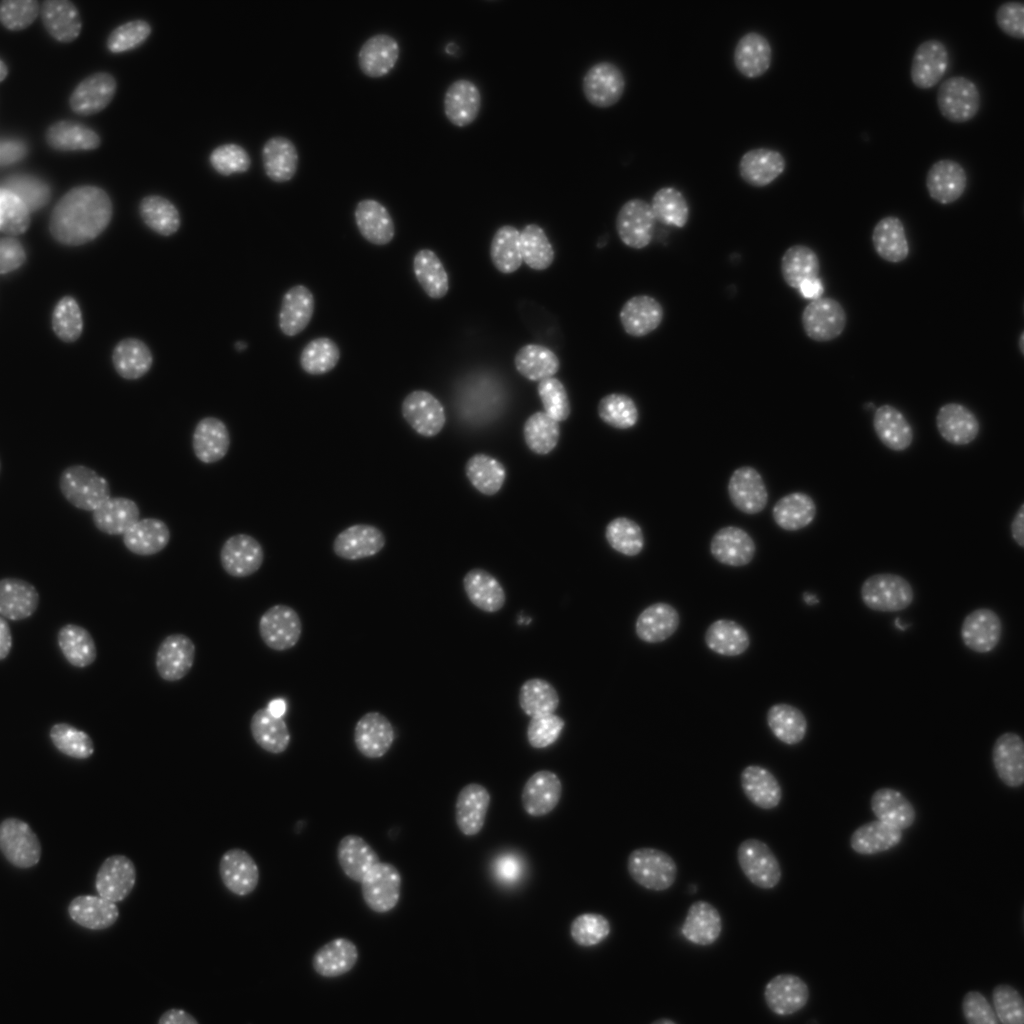

Supplement: Supplementary file 17 — Source data Fig. 8 [file 44321_2026_385_MOESM17_ESM.zip › Source Data_Figure 8/A/Replicates/GFP - Pseudo/Pseudo_4.tif]

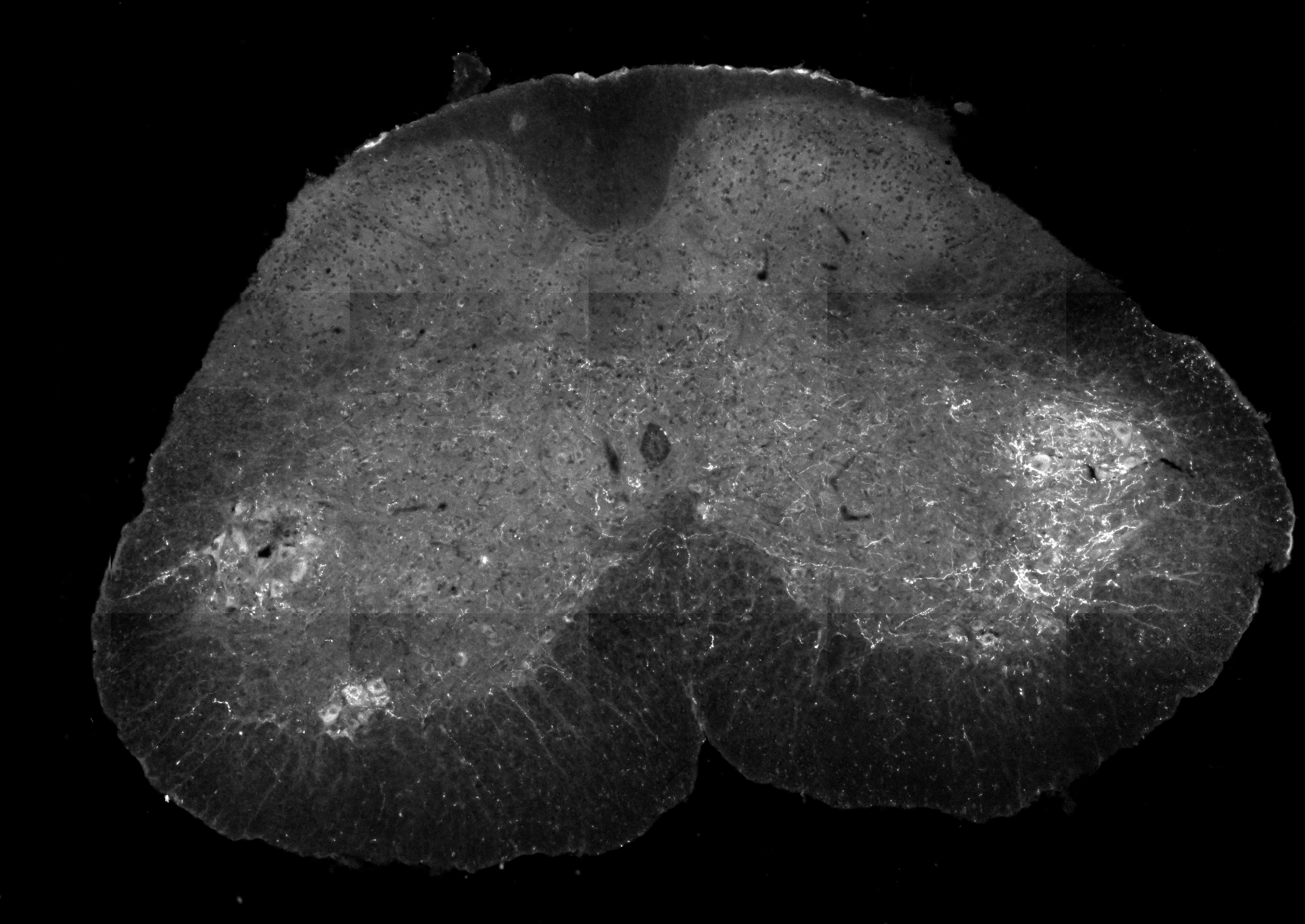

Supplement: Supplementary file 18 — Source data Fig. 9 [file 44321_2026_385_MOESM18_ESM.zip › Source Data_Figure 9/G/5HT Panobinostat.jpg]

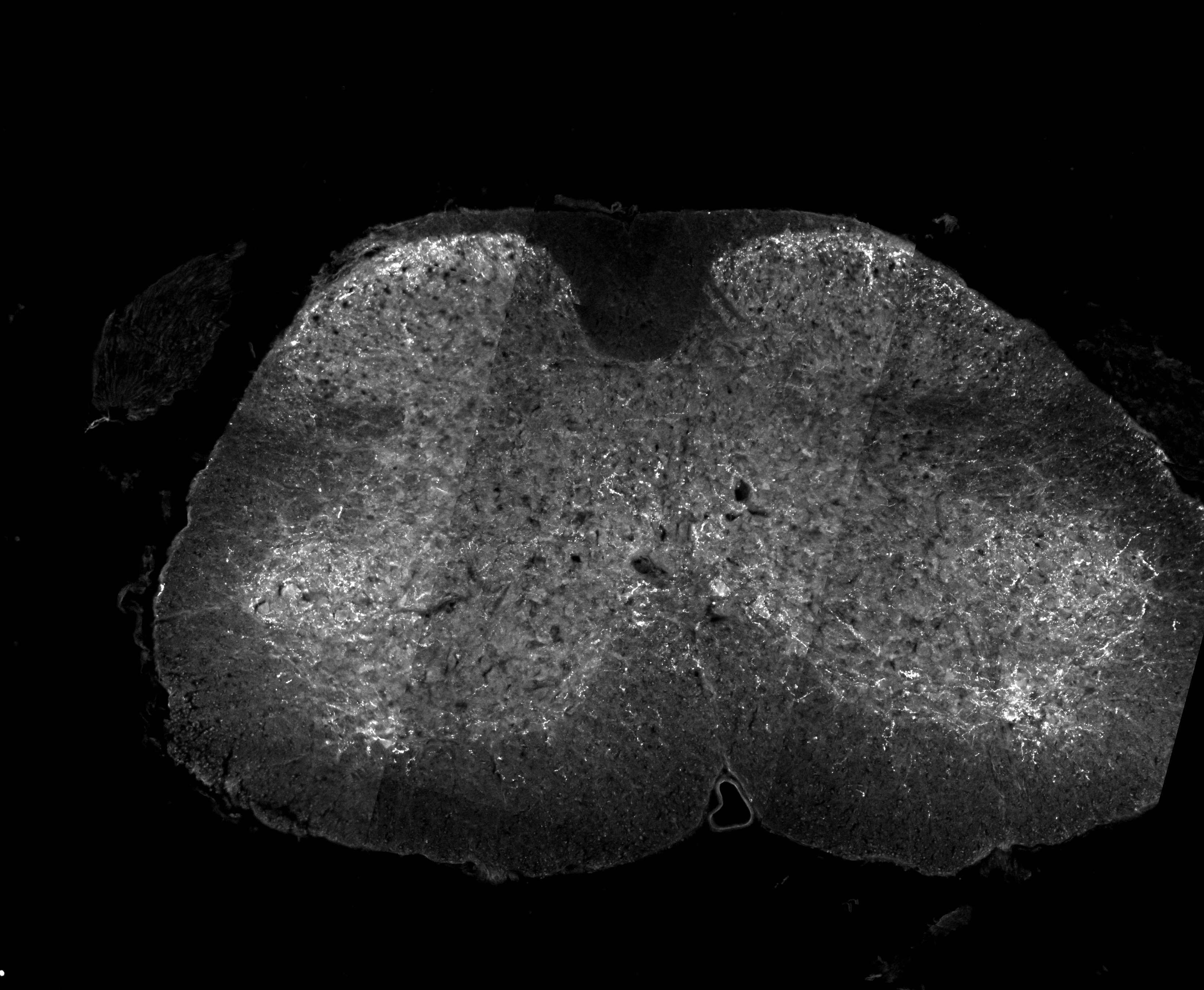

Supplement: Supplementary file 18 — Source data Fig. 9 [file 44321_2026_385_MOESM18_ESM.zip › Source Data_Figure 9/G/5HT Vechicle.jpg]

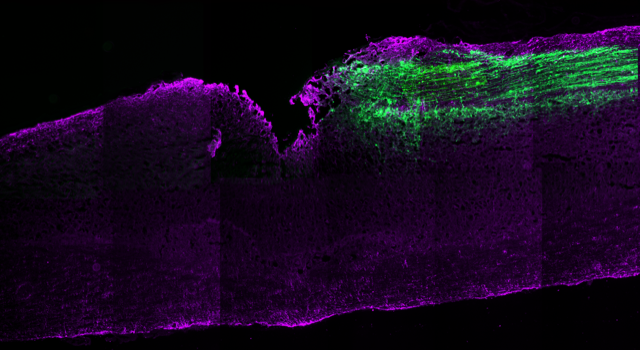

Supplement: Supplementary file 19 — Figure EV2 Source Data [file 44321_2026_385_MOESM19_ESM.tiff]
