# Supplementary material for: CITED2 is a druggable epigenetic switch coupling neuronal maturation to regenerative decline
Source: EMBO Mol Med. 2026 Feb 23;18(4):1174–201. doi: 10.1038/s44321-026-00385-w (PMC13083982; doi:10.1038/s44321-026-00385-w)
Supplement: Supplementary file 12 — Source data Fig. 3 [file 44321_2026_385_MOESM12_ESM.zip › Source Data_Figure 3/E/README.rtf]

   Immunoblotting after CITED2 over expression in F11 cell lysates.Normalised to GAPDHTop GAPDH, Bottom Cited2Lanes:Cited2-1, GFP-1, GFP-2, Cited2-2, GFP-3, CIted2-3
