# Supplementary material for: CITED2 is a druggable epigenetic switch coupling neuronal maturation to regenerative decline
Source: EMBO Mol Med. 2026 Feb 23;18(4):1174–201. doi: 10.1038/s44321-026-00385-w (PMC13083982; doi:10.1038/s44321-026-00385-w)
Supplement: Supplementary file 13 — Source data Fig. 4 [file 44321_2026_385_MOESM13_ESM.zip › Source Data_Figure 4/B/README.rtf]

Z-stack max projection representative confocal image 20xSpinal cordImage rotate, flipped, resizedBrightness, contrast adjusted, noise reducedRecoloredChannel 1 = GFAP Channel 2 = GFPChannel 3 = Dapi
