# Supplementary material for: CITED2 is a druggable epigenetic switch coupling neuronal maturation to regenerative decline
Source: EMBO Mol Med. 2026 Feb 23;18(4):1174–201. doi: 10.1038/s44321-026-00385-w (PMC13083982; doi:10.1038/s44321-026-00385-w)
Supplement: Supplementary file 14 — Source data Fig. 5 [file 44321_2026_385_MOESM14_ESM.zip › Source Data_Figure 5/README.rtf]

Data is related to a large deposited datasets.Analysis available in Datasets EV3 and EV4 and EV5 (separate files).
