# Supplementary material for: CITED2 is a druggable epigenetic switch coupling neuronal maturation to regenerative decline
Source: EMBO Mol Med. 2026 Feb 23;18(4):1174–201. doi: 10.1038/s44321-026-00385-w (PMC13083982; doi:10.1038/s44321-026-00385-w)
Supplement: Supplementary file 17 — Source data Fig. 8 [file 44321_2026_385_MOESM17_ESM.zip › Source Data_Figure 8/README.rtf]

Microscopy images of cultured embryonic DRG neuronsSource images for part of panel A provided by collaborator.Brightness adjustedFor panel A:Channel 1 = DapiChannel 2 = GFPChannel 3 = beta 3 tubularFor panel C and E:Channel 1 = DapiChannel 2 = GFPChannel 3 CBX5 or HOXD3Channel 4 = beta 3 tubular
