# Supplementary material for: CITED2 is a druggable epigenetic switch coupling neuronal maturation to regenerative decline
Source: EMBO Mol Med. 2026 Feb 23;18(4):1174–201. doi: 10.1038/s44321-026-00385-w (PMC13083982; doi:10.1038/s44321-026-00385-w)
Supplement: Supplementary file 17 — Source data Fig. 8 [file 44321_2026_385_MOESM17_ESM.zip › Source Data_Figure 8/A/MS group collaboration - cited2 results.pptx]

## Slide 1
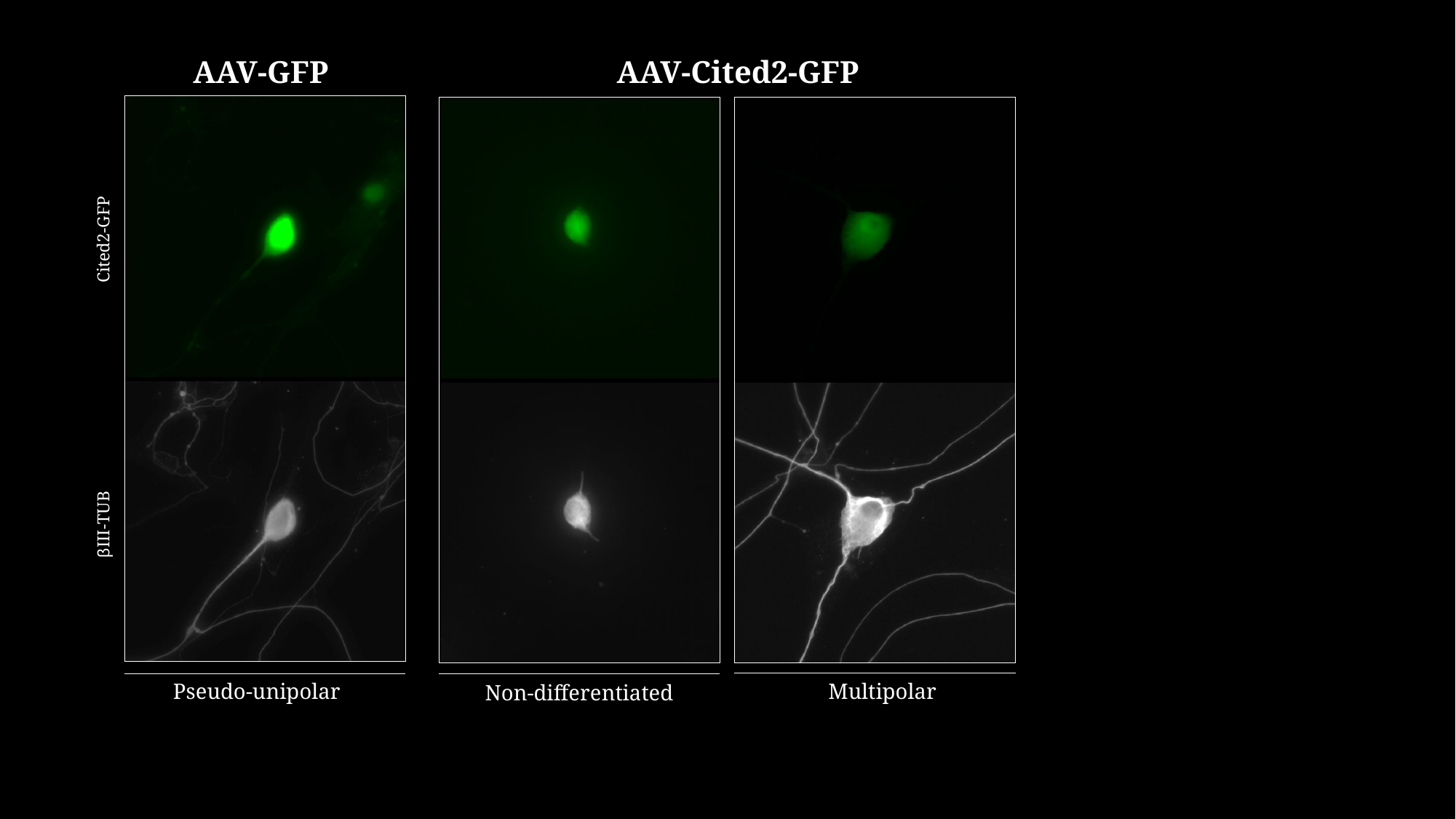

AAV-GFP
AAV-Cited2-GFP
βIII-TUB
Cited2-GFP
βIII-TUB
Multipolar
Pseudo-unipolar
Non-differentiated
