# Supplementary material for: CITED2 is a druggable epigenetic switch coupling neuronal maturation to regenerative decline
Source: EMBO Mol Med. 2026 Feb 23;18(4):1174–201. doi: 10.1038/s44321-026-00385-w (PMC13083982; doi:10.1038/s44321-026-00385-w)
Supplement: Supplementary file 18 — Source data Fig. 9 [file 44321_2026_385_MOESM18_ESM.zip › Source Data_Figure 9/README.rtf]

Spinal cord confocal images. Stitched, max z stack projection.Brightness  and contrast adjustedFor panel B:Recoloured Channel 1 = GFAPChannel 2 = Dextran For panel GLUTs inverted
