# Supplementary material for: CITED2 is a druggable epigenetic switch coupling neuronal maturation to regenerative decline
Source: EMBO Mol Med. 2026 Feb 23;18(4):1174–201. doi: 10.1038/s44321-026-00385-w (PMC13083982; doi:10.1038/s44321-026-00385-w)
Supplement: Supplementary file 20 — Expanded View Figures [file 44321_2026_385_MOESM20_ESM.pdf]

Expanded View Figures

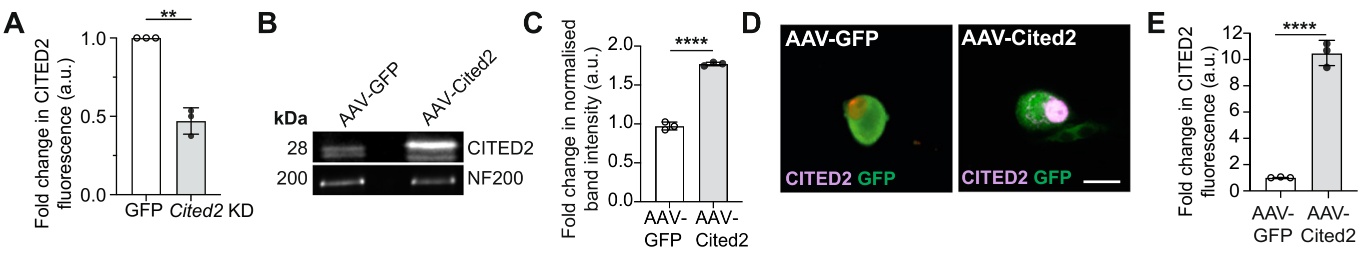

Figure EV1. Cited2 downregulation and overexpression.

(**A**) Quantification of Cited2 knockdown in cultured DRG neurons (*t*-test, $p = 0.0092$, $n = 3$ independent biological replicates, about 50 cells per replicate). (**B**, **C**) Confirmation and quantification of CITED2 overexpression efficiency following AAV-GFP or AAV-Cited2-GFP in DRG cultured neurons using immunoblotting analysis. Band intensity normalized against NF200 (*t*-test, $p = 1.492 \times 10^{-5}$, $n = 3$ independent biological replicates). (**D**, **E**) Confirmation and quantification of CITED2 overexpression (magenta) in GFP^{+} DRG cultured neurons after addition of AAV-GFP or AAV-Cited2-GFP (*t*-test, $p = 4.516 \times 10^{-5}$, $n = 3$ independent biological replicates, about 50 cells per replicate). Scale bar: 20 µm. *$p < 0.05$, **$p < 0.01$, ***$p < 0.001$, and ****$p < 0.0001$. ns not significant. All error bars shown as standard deviation (SD).

© The Author(s)

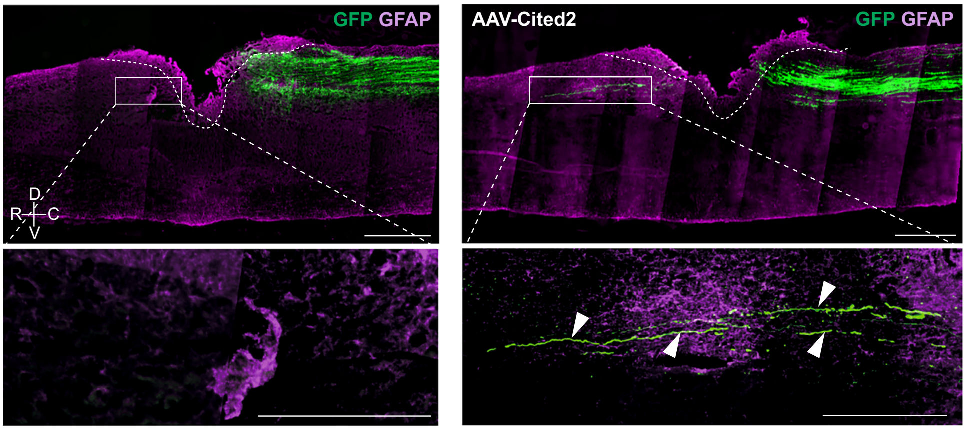

Figure EV2. Additional representative micrographs of AAV-GFP or AAV-Cited2-GFP overexpression.

AAV-Cited2-GFP promoted axon growth into and past the lesion site (green, white arrows) 6 weeks post-SCI. GFAP (magenta) was used to determine the lesion site (white dotted line). Scale bar: 200 µm. Scale bar zoomed inset: 100 µm. Source data are available online for this figure.

© The Author(s)

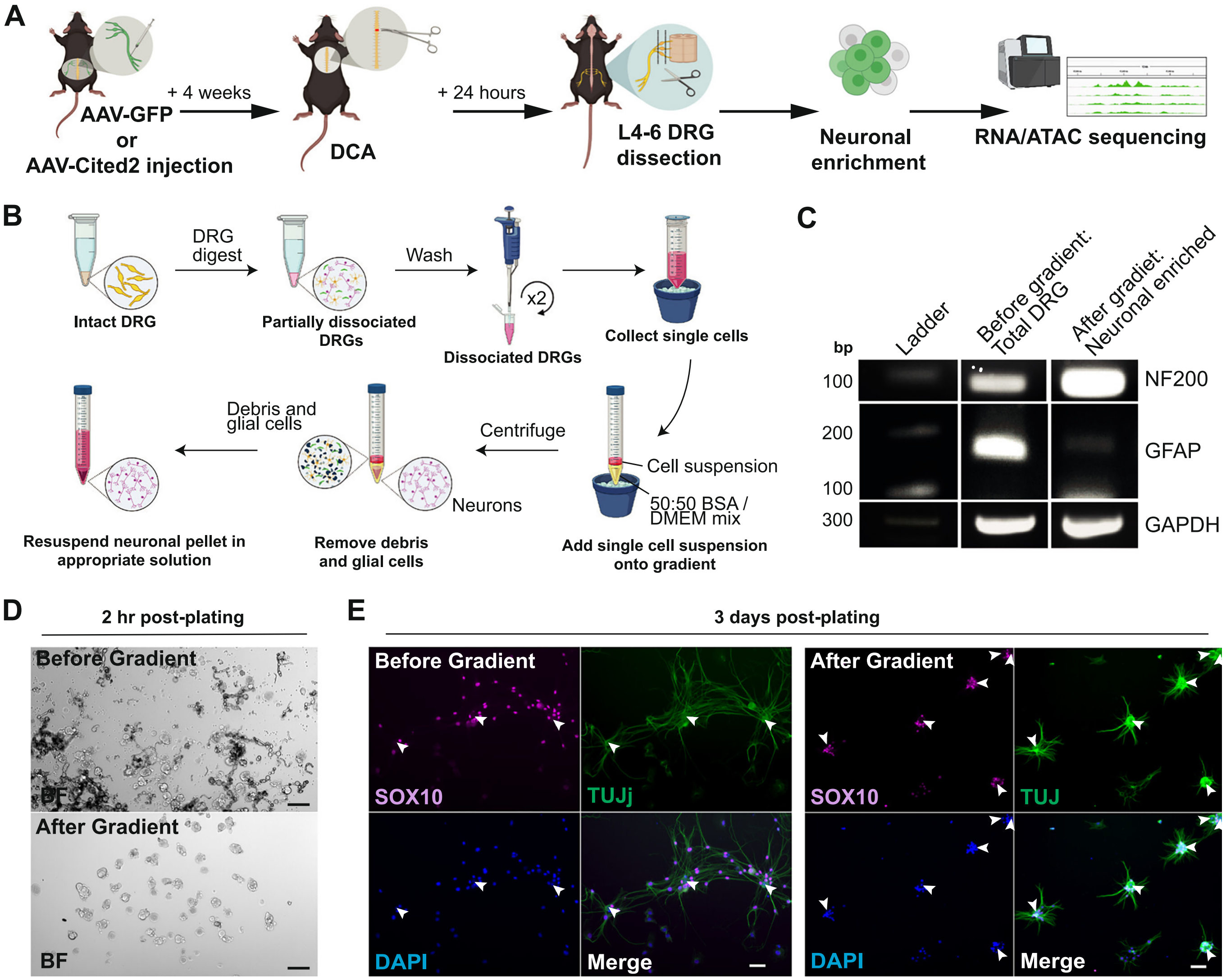

Figure EV3. Confirmation of neuronal enrichment following gradient separation.

(**A**, **B**) Experimental design (made with BioRender). (**C**) mRNA expression as determined by reverse transcription (RT)-PCR with neuronal markers (NF200) or glial markers (GFAP) before and after neuronal gradient separation. GAPDH was used to normalize band intensity. (**D**) Representative brightfield micrographs of DRG cells before and after neuronal gradient. (**E**) SOX10 (magenta) and beta 3 tubulin (TUJ, green) immunostaining in DRG cells before and after neuronal gradient. Scale bar: 50 µm.

© The Author(s)

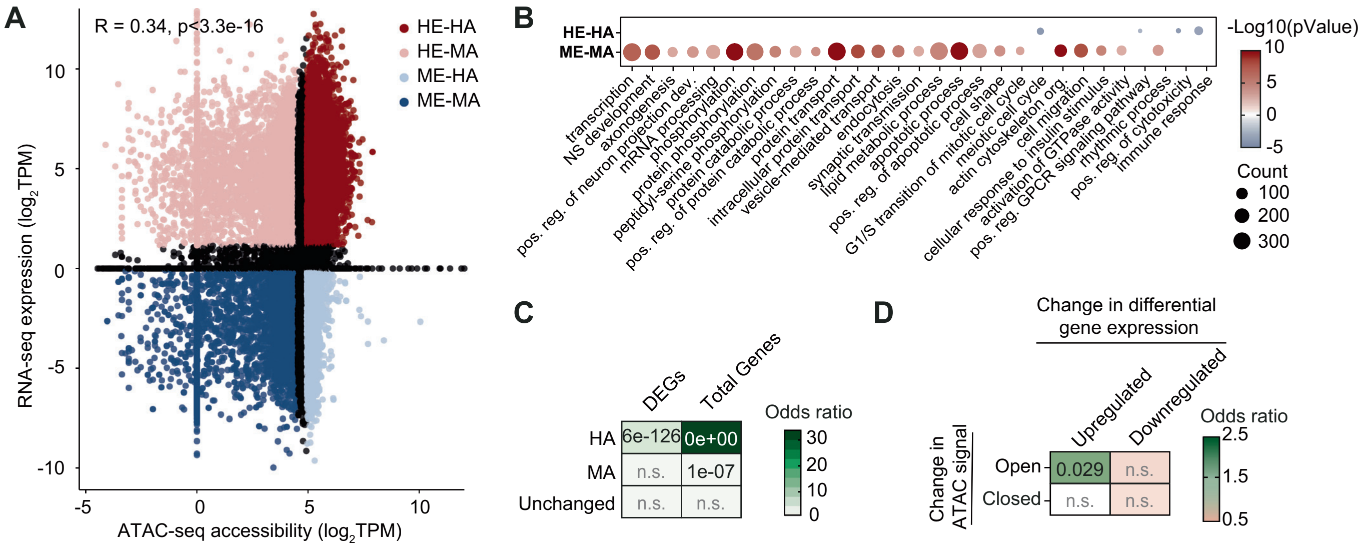

Figure EV4. Gene expression and chromatin accessibility after Cited2 overexpression and SCI.

(**A**) Correlation analysis between gene expression (RNA-seq) and chromatin accessibility (ATAC-seq) following Cited2 overexpression [Spearman correlation *p* value < 3.714×10^{-7}, $R^2 = 0.34$] using log2 of normalized TPM. Genes are split into four categories: highly expressed and highly accessible (HE-HA 7582 genes) if their normalized TPM is higher than the 75th percentile of the data; low-to-medium expression and low-to-medium accessibility (ME-MA 6420 genes) if their TPM is lower than the 50th percentile of the data; highly expressed and low-to-medium accessibility (HE-MA 4336 genes); and low-to-medium expression and high accessibility (ME-HA 2396 genes). Black dots indicate genes that do not fall into any category. (**B**) GO and KEGG analysis of HA-HE or ME-MA genes. *P* values are calculated using a modified Fisher's exact test as described in DAVID (Huang et al, 2009). (**C**) Odds ratio and Fisher's exact tests between differentially expressed (DEGs, $p < 0.05$) or non-differentially expressed (Total genes, $p > 0.05$) genes and genes showing high accessibility (HA), low-to-medium accessibility (MA), or unchanged accessibility (UA). (**D**) Odds ratio and Fisher's exact tests between changes in differential gene expression (DEGs, $p < 0.05$) and changes in chromatin accessibility (ATAC signal, $p < 0.05$). Color represents correlation (odds ratio) while numbers represent significance (Fisher's exact test).

 © The Author(s)

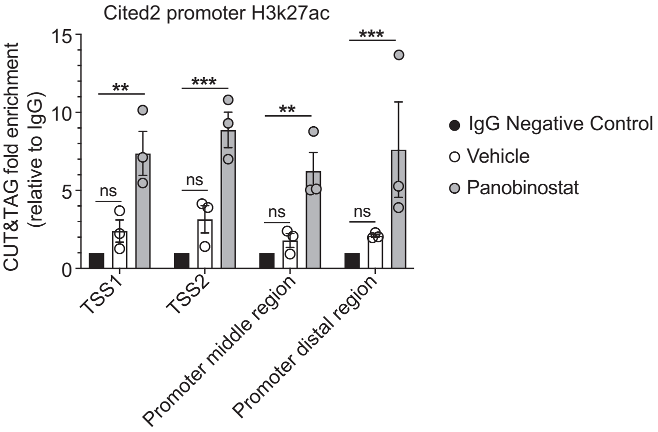

Figure EV5. Panobinostat increases H3k27ac occupancy at the *Cited2* promoter in adult DRG neurons.

CUT&Tag qPCR quantification of H3k27ac at two TSS sites (TSS1 and TSS2), the middle promoter region, and the distal promoter region. Data were expressed relative to the IgG-negative control. Two-way ANOVA with Dunnett's post hoc test (Group: $f(2,24) = 36$, $p = 5.010 \times 10^{-8}$; Region: $f(3,24) = 0.6$, $p = 0.5661$; Interaction score: $f(6,24) = 0.2$, $p = 0.9601$. Displayed is the Treatment p value. IgG vs Panobinostat—TSS1, $p = -0.0011$; TSS2, $p = 0.0001$; middle promoter region, $p = 0.0063$; distal promoter region, $p = 0.0008$; IgG vs vehicle, not significant for all regions. $n = 3$ independent biological replicates per group. $^{*}p < 0.05$, $^{**}p < 0.01$, $^{***}p < 0.001$, and $^{****}p < 0.0001$. ns not significant. All error bars shown as standard deviation (SD).

© The Author(s)
